# HNRNPD Induces Radioresistance in Nasopharyngeal Carcinoma by Sequestering *GRAMD4* mRNA in Stress Granules

**DOI:** 10.1002/advs.202521038

**Published:** 2026-05-10

**Authors:** Yingzi Li, Tong Xiang, Yuanyuan Liu, Desheng Weng, Jingjing Zhao, Hao Chen, Yan Tang, Songzuo Xie, Hao Zhang, Jun Luo, Xinyi Yang, Qiuzhong Pan, Muping Di, Jianchuan Xia

**Affiliations:** ^1^ State Key Laboratory of Oncology in South China Guangdong Key Laboratory of Nasopharyngeal Carcinoma Diagnosis and Therapy Guangdong Provincial Clinical Research Center for Cancer Sun Yat‐sen University Cancer Center Guangzhou China; ^2^ Department of Biotherapy Sun Yat‐sen University Cancer Center Guangzhou China; ^3^ Department of Experimental Research Sun Yat‐sen University Cancer Center Guangzhou Guangdong China; ^4^ Department of Medical Oncology The Third Affiliated Hospital of Sun Yat‐Sen University Guangzhou China; ^5^ Department of Nuclear Medicine Sun Yat‐sen University Cancer Center Guangzhou China; ^6^ Department of Radiation Oncology Nanfang Hospital Southern Medical University Guangzhou China

**Keywords:** apoptosis, *GRAMD4*, HNRNPD, nasopharyngeal carcinoma, radioresistance, stress granules

## Abstract

Radiotherapy resistance remains a major obstacle in nasopharyngeal carcinoma (NPC). Stress granules (SGs), dynamic cytoplasmic ribonucleoprotein condensates, have been linked to therapy resistance, but their role in NPC radioresistance remains unclear. Here, we show that SGs are key mediators of NPC radioresistance. Radioresistant NPC cells displayed markedly enhanced SG formation in a dose‐ and time‐dependent manner, whereas pharmacological inhibition with ISRIB or genetic disruption of G3BP1 significantly sensitized cells to irradiation in vitro and in vivo. Integrated transcriptomic and proteomic analyses identified heterogeneous nuclear ribonucleoprotein D (HNRNPD) as a critical SG‐associated RNA‐binding protein upregulated in resistant cells and associated with poor prognosis. Functionally, HNRNPD promoted radioresistance by suppressing apoptosis, whereas its depletion restored radiosensitivity. Mechanistically, HNRNPD underwent RNA‐dependent phase separation through its C‐terminal intrinsically disordered region and interacted with G3BP1 to facilitate SG assembly. Irradiation promoted cytoplasmic accumulation of HNRNPD, while the p37 isoform preferentially bound G3BP1 and functionally drove SG formation. HNRNPD further bound *GRAMD4* mRNA via its RRM1 domain and sequestered it in SG‐associated compartments, thereby repressing GRAMD4 translation, suppressing mitochondrial apoptosis, and promoting survival. Restoration of GRAMD4 abrogated HNRNPD‐induced radioresistance. Together, these findings establish the HNRNPD–SG–GRAMD4 axis as a key determinant of NPC radioresistance and a potential therapeutic target for radiosensitization.

## Graphical abstract

HNRNPD promotes radioresistance in nasopharyngeal carcinoma by enhancing stress granule assembly and sequestering GRAMD4 mRNA. This suppresses GRAMD4 translation and inhibits mitochondrial apoptosis. Targeting the integrated stress response with ISRIB restores GRAMD4 expression and sensitizes tumors to radiotherapy, revealing a translational control mechanism underlying radioresistance.

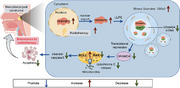

## 1 Introduction

Nasopharyngeal carcinoma (NPC) is a cancer of the head and neck that is most prevalent in Southeast Asia, particularly in southern China [1]. Owing to its anatomical location and inherent radiosensitivity, radiotherapy remains the primary treatment for NPC patients [2]. However, approximately 10–20% of patients experience local recurrence after curative radiotherapy, and those with recurrent NPC (rNPC) face significantly worse survival outcomes [3]. Notably, approximately 90% of local recurrent lesions arise within areas that previously received high radiation doses [4]. Although hyperfractionated intensity‐modulated radiotherapy (IMRT) has been employed to improve survival in patients with rNPC, nearly 49% of patients who undergo reirradiation still experience further recurrence [5]. These observations highlight radiation resistance as a major cause of local recurrence in NPC and underscore the urgent need to elucidate the molecular mechanisms of radioresistance and develop effective radiosensitization strategies.

Stress granules (SGs) are dynamic ribonucleoprotein aggregates that form in response to cellular stressors such as oxidative stress, hypoxia, or heat shock [6]. SGs, which are composed mainly of mRNA and RNA‐binding proteins (RBPs), serve as adaptive structures that increase cell survival under adverse conditions [7]. SGs rapidly assemble in the cytoplasm upon stress exposure and disassemble once the stress is relieved, reflecting their reversible and dynamic nature [6]. In tumors, SGs help cancer cells adapt to hostile microenvironments and promote treatment resistance. For example, in gastric cancer, overexpression of the SG nucleator G3BP1 is correlated with cisplatin resistance, as SG assembly suppresses chemotherapy‐induced apoptosis and enhances tumor cell survival [8]. In colorectal cancer, m6A‐mediated stabilization of LIMK1 enhances SG formation and confers resistance to 5‐fluorouracil [9]. These findings indicate that SGs act as hubs for mRNA sequestration and translational repression, thereby promoting cell survival. However, their role in NPC radioresistance remains poorly defined.

RBPs are essential components of SGs and regulate the fate of mRNAs under stress conditions. For example, during endoplasmic reticulum (ER) stress, Ataxin‐2 sequesters *Xbp1* mRNA within SGs, preventing its translation until Fbxo42‐mediated Ataxin‐2 degradation releases the transcript to trigger UPR‐associated cell death [10]. Sumoylated PABPC1 recruits U‐rich mRNAs (such as *FUNDC1* and *BNIP3L*) to SGs, protecting them from degradation and thereby enhancing cancer cell survival [11]. In addition to mRNA regulation, RBPs can also drive SG assembly through liquid–liquid phase separation (LLPS). G3BP1, a core SG nucleator, dimerizes via its NTF2L domain, whereas its C‐terminal RNA‐binding domain (RBD) and intrinsically disordered region (IDR) promote LLPS to initiate SG assembly [6]. Other RBPs, including TIA1, FUS, and HNRNPA2B1, contribute to SG regulation via similar mechanisms [12, 13, 14]. Notably, HNRNP family members, such as HNRNPK, have been implicated in radiosensitization by modulating the DNA damage response and gene expression in head and neck squamous cell carcinoma (HNSCC) [15]. However, whether heterogeneous nuclear ribonucleoprotein D (HNRNPD), an important HNRNP family member involved in mRNA stability and translation, regulates SG dynamics and thereby contributes to NPC radioresistance remains unknown.

In this study, we show that SG formation is markedly enhanced in radioresistant NPC cells and that either pharmacological inhibition or genetic disruption of SGs sensitizes these cells to irradiation. We identify HNRNPD as a critical regulator of SG dynamics and further demonstrate that the p37 isoform preferentially interacts with G3BP1 to promote SG assembly. Mechanistically, HNRNPD suppresses apoptosis by sequestering *GRAMD4* mRNA in SG‐associated compartments and repressing its translation, thereby enhancing NPC radioresistance. These findings uncover a previously unrecognized mechanism of radioresistance in NPC and suggest that targeting SGs or the HNRNPD axis may represent a promising strategy to improve the efficacy of radiotherapy.

## 2 Results

### 2.1 SG Formation Promotes Radioresistance in NPC

**FIGURE 1:**
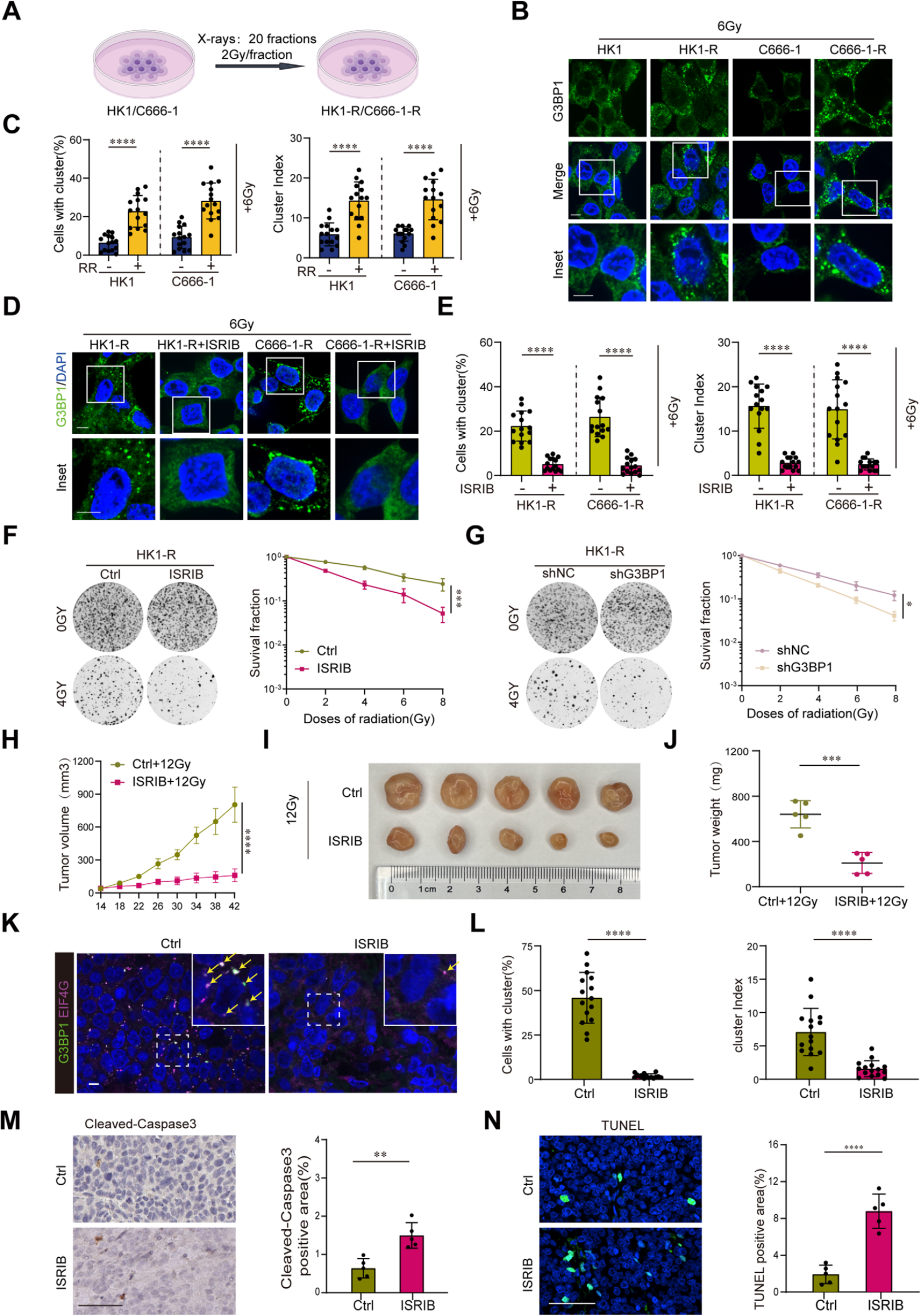
Stress granule formation promotes radioresistance in nasopharyngeal carcinoma. (A) Schematic of the establishment of radioresistant NPC cell lines (HK1‐R and C666‐1‐R) from parental HK1 and C666‐1 cells via fractionated irradiation at a total dose of 40 Gy. (B) Representative confocal microscopy images of stress granules (SGs) visualized by immunofluorescence (IF) staining for the SG core marker G3BP1 [14] in parental and radioresistant NPC cells at 9 h after 6 Gy irradiation. Nuclei were stained with DAPI (blue). Scale bar, 5 µm. Insets show magnified views of the demarcated regions. Scale bar of the inset, 5 µm. (C) Quantification of the percentage of SG‐positive cells and the SG cluster index (mean number of SGs per positive cell). The data are presented as the means ± SDs (*n* = 15 image fields per condition from three independent experiments). (D) Representative confocal microscopy images of G3BP1 in HK1‐R and C666‐1‐R cells after 6 Gy irradiation, followed by treatment with or without 2 µM ISRIB for 4 h. Scale bar, 5 µm. Scale bar of the inset, 5 µm. (E) Quantification of the percentage of SG‐positive cells and the SG cluster index from (D). The data are shown as the means ± SDs (*n* = 15 image fields per condition from three independent experiments). (F) Left, schematic of the experimental timeline for the colony formation assays. Right, clonogenic survival curves for HK1‐R cells treated with or without ISRIB following exposure to the indicated doses of radiation. The data represent the means ± SDs of three independent experiments. (G) Left, schematic of the experimental timeline. Right, clonogenic survival curves for HK1‐R cells transfected with control or G3BP1‐targeting shRNA after exposure to the indicated radiation doses. The data represent the means ± SDs of three independent experiments. (H) Tumor growth curves of HK1‐R xenografts in NCG mice subjected to the indicated treatments (RT, 2 Gy/fraction, 6 fractions). Data represent the means ± SDs (*n* = 5 mice per group). (I) Representative images of excised tumors at the experimental endpoint (Day 42). (J) Tumor weights on Day 42. Data represent the means ± SDs (*n* = 5 tumors per group). (K) Representative IF micrographs of G3BP1 [14], eIF4G (purple), and DAPI (blue) in tumor sections from the indicated treatment groups. Yellow arrows indicate SG clusters (G3BP1 and eIF4G costaining). Scale bar, 50 µm. (L) Quantification of the percentage of SG‐positive cells and the SG cluster index from (K). Data are shown as the means ± SDs (*n* = 15 image fields per condition from five independent tumors). (M, N) Quantification of the areas positively stained for cleaved caspase‐3 (M) and TUNEL (N) in tumor sections from the indicated groups. Scale bar, 50 µm. Data represent the means ± SDs (*n* = 5 independent tumors). *p < 0.05; **p < 0.001; ***p < 0.0001; statistical significance was determined by two‐way ANOVA followed by Šídák's multiple‐comparisons test (F–H) and two‐sided unpaired Student's *t*‐test (C, E, J, L–N).

To investigate the relationship between SGs and radiosensitivity, we established radiation‐resistant NPC cell lines (HK1‐R and C666‐1‐R) by subjecting parental HK1 and C666‐1 cells to fractionated irradiation to a total dose of 40 Gy (Figure 1A) [16]. Colony formation assays following exposure to graded single doses of radiation (0–8 Gy) showed that both HK1‐R and C666‐1‐R cells exhibited significantly increased clonogenic survival compared with their parental counterparts (Supplementary Figure S1A,B). Compared with the HK1/HK1‐R pair, the C666‐1/C666‐1‐R pair displayed lower overall survival after irradiation, indicating greater intrinsic radiosensitivity.

Confocal microscopy revealed robust SG formation (0.1–2‐µm puncta) after irradiation, with markedly higher levels in resistant cells following 6 Gy treatment (Figure 1B,C). SG assembly increased in a dose‐ and time‐dependent manner, peaking at approximately 9 h after 6 Gy irradiation (Supplementary Figure S1C–J). Time‐lapse live‐cell imaging of G3BP1‐EGFP‐transfected HK1‐R cells further showed that radiation‐induced SGs were highly dynamic and underwent continuous assembly and disassembly over time (Supplementary Video S1). Thus, although the proportion of SG‐positive cells at any single time point peaked at only about 30% after 6 Gy irradiation, cumulative observation over the imaging period revealed that more than half of the cells formed transient G3BP1‐positive granules at least once, indicating that snapshot‐based measurements underestimate the overall extent of SG engagement. Notably, C666‐1‐R cells showed earlier SG induction and responded to lower radiation doses, suggesting a lower threshold for radiation‐triggered stress responses.

To further assess the functional contribution of SGs to radioresistance, we used ISRIB, which antagonizes the integrated stress response downstream of phosphorylated eIF2α and thereby suppresses SG assembly [17, 18]. Pharmacological inhibition with ISRIB significantly reduced both the proportion of SG‐positive cells and the SG cluster index (Figure 1D,E), accompanied by increased radiosensitivity in colony formation assays (Figure 1F; Supplementary Figure S1K). Similarly, genetic disruption of the SG core protein G3BP1 also increased radiosensitivity (Figure 1G; Supplementary Figure S1L). Consistent with the mechanism of ISRIB, radioresistant HK1‐R and C666‐1‐R cells displayed elevated basal and irradiation‐induced p‐eIF2α levels compared with their parental counterparts, whereas total eIF2α remained largely unchanged (Supplementary Figure S2A,B). Time‐course analysis in HK1‐R cells further showed that p‐eIF2α increased after irradiation, peaked at 9–12 h, and was followed by the later accumulation of cleaved caspase‐3 (Supplementary Figure S2C). Consistent with this temporal pattern, flow cytometric analysis of cell death in HK1‐R cells showed no significant increase at 9 h after 6 Gy irradiation compared with the 0 Gy control at 72 h, whereas a marked increase was observed at 72 h after 6 Gy irradiation (Supplementary Figure S2D), supporting the interpretation that SG‐associated stress signaling occurs earlier than overt apoptotic cell death.

In vivo, combination treatment with ISRIB and radiotherapy markedly suppressed HK1‐R xenograft growth, resulting in reduced tumor volume and tumor weight, together with decreased intratumoral SG formation and increased apoptotic markers, including cleaved caspase‐3 and TUNEL‐positive cells (Figure 1H–N). Importantly, ISRIB did not cause overt systemic toxicity, as indicated by stable body weight, no obvious histopathological abnormalities in the heart, liver, spleen, lung, or kidney, and no significant changes in hematological or serum biochemical parameters (Supplementary Figure S2E–H).

To further evaluate the therapeutic effect in an immune‐relevant setting, we established a huPBMC‐NCG model, in which successful human immune‐cell engraftment was confirmed by the progressive increase in hCD45+ cells in peripheral blood from day 7 to day 21 (Supplementary Figure S3A). In this humanized model, ISRIB combined with radiotherapy again significantly reduced HK1‐R tumor growth and tumor weight (Supplementary Figure S3B–D). Flow cytometric analysis of tumor‐infiltrating human immune cells showed no significant change in the proportion of CD8+ cells among hCD45+ cells or in Granzyme B+ cells within the CD8+ population (Supplementary Figure S3E–G), suggesting that the antitumor effect of ISRIB in this setting mainly reflects tumor‐intrinsic radiosensitization rather than a major alteration in CD8+ T‐cell infiltration or effector status.

Furthermore, CRISPR‐mediated G3BP1 knockout in HK1‐R cells, confirmed by immunoblotting, markedly reduced clonogenic survival after irradiation, whereas G3BP1 re‐expression restored radioresistance (Supplementary Figure S4A, B). This genetic rescue pattern was recapitulated in vivo, where G3BP1 knockout suppressed xenograft growth and reduced tumor weight after radiotherapy, while G3BP1 re‐expression partially reversed these effects (Supplementary Figure S4C–E).

Collectively, these findings demonstrate that SG assembly is a key determinant of NPC radioresistance and that both pharmacologic and genetic disruption of SGs restore radiosensitivity.

### 2.2 HNRNPD is Upregulated in Radioresistant NPC and is Associated with Poor Prognosis

**FIGURE 2:**
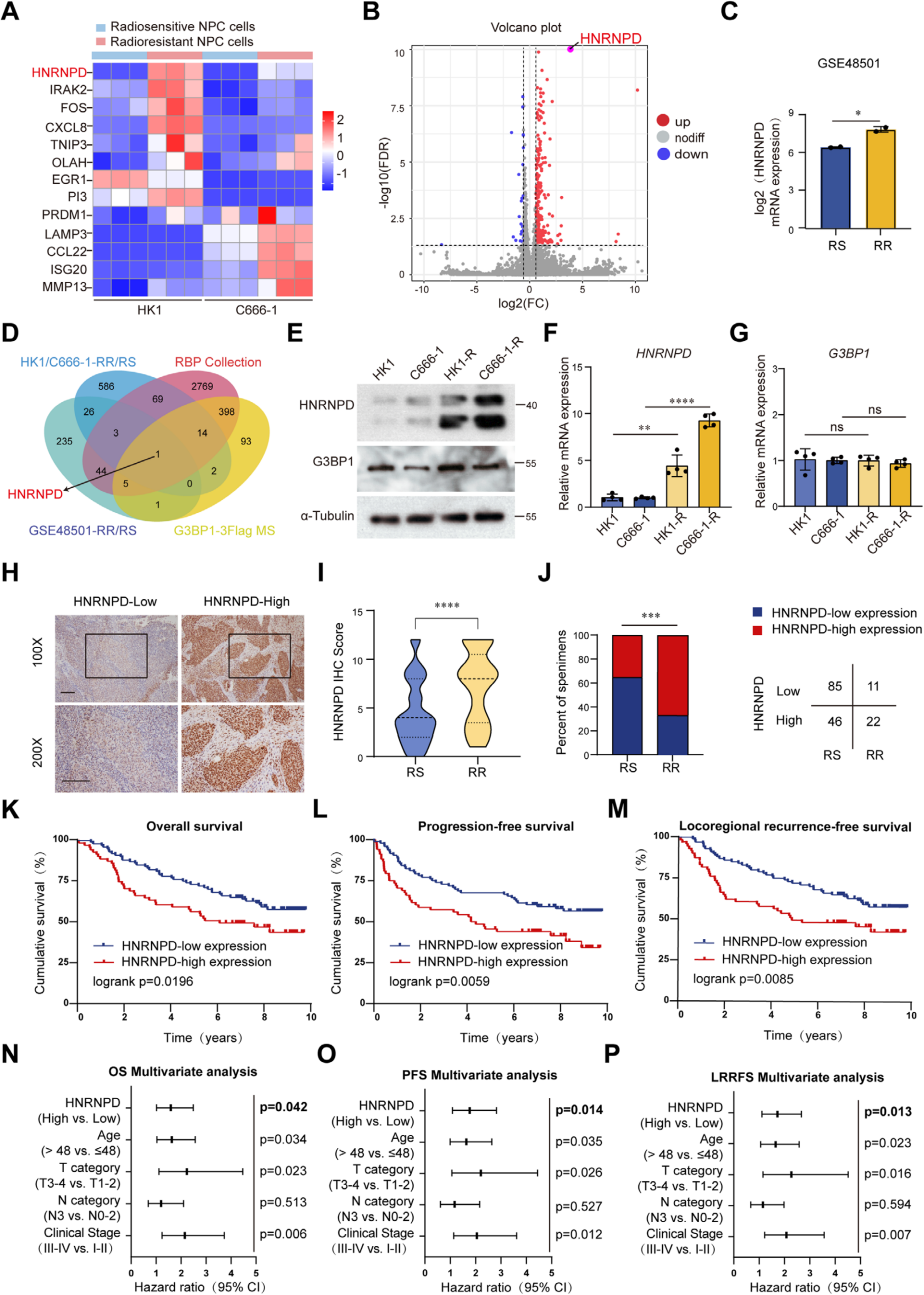
HNRNPD is upregulated in radioresistant NPC and is associated with poor prognosis. (A) Heatmap of RNA‐sequencing data from radiosensitive (parental) and radioresistant (R) HK1 and C666‐1 cell lines. (B) Volcano plot displaying genes that are differentially expressed between HK1 and HK1‐R cells. (C) Analysis of *HNRNPD* mRNA expression in radioresistant (*n* = 2) versus radiosensitive (*n* = 2) NPC tissues from the GEO dataset GSE48501. The data are presented as the means ± SDs. (D) Venn diagram identifying HNRNPD as a common candidate through the integration of differentially expressed genes from the HK1/HK1‐R and C666‐1/C666‐1‐R pairs, mass spectrometry (MS) of G3BP1 interactors, an RNA‐binding protein (RBP) database, and the NPC radioresistance dataset GSE48501. (E) Western blot analysis of HNRNPD and G3BP1 protein levels in the indicated cell lines. (F, G) qRT‒PCR analysis of *HNRNPD* (F) and *G3BP1* (G) mRNA levels in the indicated cell lines. The data are presented as the means ± SDs of three independent experiments. (H) Representative immunohistochemistry (IHC) images of NPC tissue samples with low (left) and high (right) HNRNPD expression. Scale bar, 100 µm. The bottom panels show magnified insets. (I) Quantification of IHC scores for HNRNPD in radiosensitive and radioresistant NPC tissues. The data are presented as the means ± SDs (*n* = 131 RS, *n* = 33 RR). (J) Contingency table analysis showing the correlation between the HNRNPD expression level and clinical radioresistance status. (K‒M) Kaplan‒Meier survival curves for patients with NPC stratified by high or low HNRNPD IHC expression, showing overall survival (OS, K), progression‐free survival (PFS, L), and locoregional relapse‐free survival (LRRFS, M). (N‒P) Cox regression analysis of potential prognostic factors, including HNRNPD levels and other clinical characteristics. ns, not significant; *p < 0.05, **p < 0.01, ***p < 0.001, ****p < 0.0001; statistical significance was determined by two‐sided unpaired Student's *t*‐test (F, G, I), χ^2^ test (J), and log‐rank test (K–M). NPC, nasopharyngeal carcinoma; RS, radiosensitive; RR, radioresistant.

Given the crucial role of RBPs in SG assembly and function [6], we investigated the mechanisms through which SGs contribute to tumor radioresistance. We performed RNA sequencing on radiosensitive and radioresistant NPC cells (Figure 2A,B) combined with proteomics to identify proteins that interact with G3BP1 (Supplementary Figure S5A,B). By integrating these data with an RBP database (http://research.gzsys.org.cn/eurbpdb2/index.html) and an NPC radioresistance dataset (GSE48501, Figure 2C), we identified HNRNPD as a candidate SG regulator (Figure 2D). In radioresistant NPC cell lines, HNRNPD was consistently overexpressed at both mRNA and protein levels, whereas the level of G3BP1 remained unchanged (Figure 2E–G). Therefore, we hypothesized that HNRNPD may promote radioresistance by facilitating SG formation rather than by upregulating G3BP1 expression in radioresistant NPC cell lines.

The Cancer Genome Atlas (TCGA) and Gene Expression Omnibus (GEO) dataset analyses revealed that HNRNPD expression was significantly upregulated in various cancers, including HNSCC, as well as in tumor samples, compared with that in paired normal tissues (Supplementary Figure S5C–F). To further assess the clinical significance of HNRNPD in NPC, we performed immunohistochemical staining in a cohort of 164 NPC specimens. The baseline clinicopathological characteristics of this cohort and the tumor‐specific HNRNPD expression patterns are summarized in Supplementary Table S1. According to staining intensity, NPC tissues were classified as negative, weak, moderate, or strong for HNRNPD expression (Supplementary Figure S5G). The relationships between HNRNPD expression and clinicopathological features are presented in Supplementary Table S2, which showed that elevated HNRNPD expression was significantly associated with radioresistance (Figure 2H–J). Kaplan–Meier analysis further demonstrated that high HNRNPD expression was significantly associated with worse overall survival (OS), progression‐free survival (PFS), and locoregional relapse‐free survival (LRRFS) (Figure 2K–M). To determine whether this association was independent of other clinicopathological variables, we performed univariate and multivariate Cox regression analyses, the results of which are summarized in Supplementary Table S3. These analyses identified HNRNPD as an independent adverse prognostic factor for OS, PFS, and LRRFS in NPC (Figure 2N–P). Collectively, these findings indicate that HNRNPD is upregulated in radioresistant NPC and is closely associated with poor clinical outcome.

### 2.3 HNRNPD Promotes Radioresistance in NPC Cells In Vitro and In Vivo

To further define the role of HNRNPD in NPC radiosensitivity, we generated HK1 and C666‐1 cells stably overexpressing HNRNPD, and HK1‐R and C666‐1‐R cells with stable HNRNPD knockdown, which was confirmed by immunoblotting (Supplementary Figure S6A–D).

**FIGURE 3:**
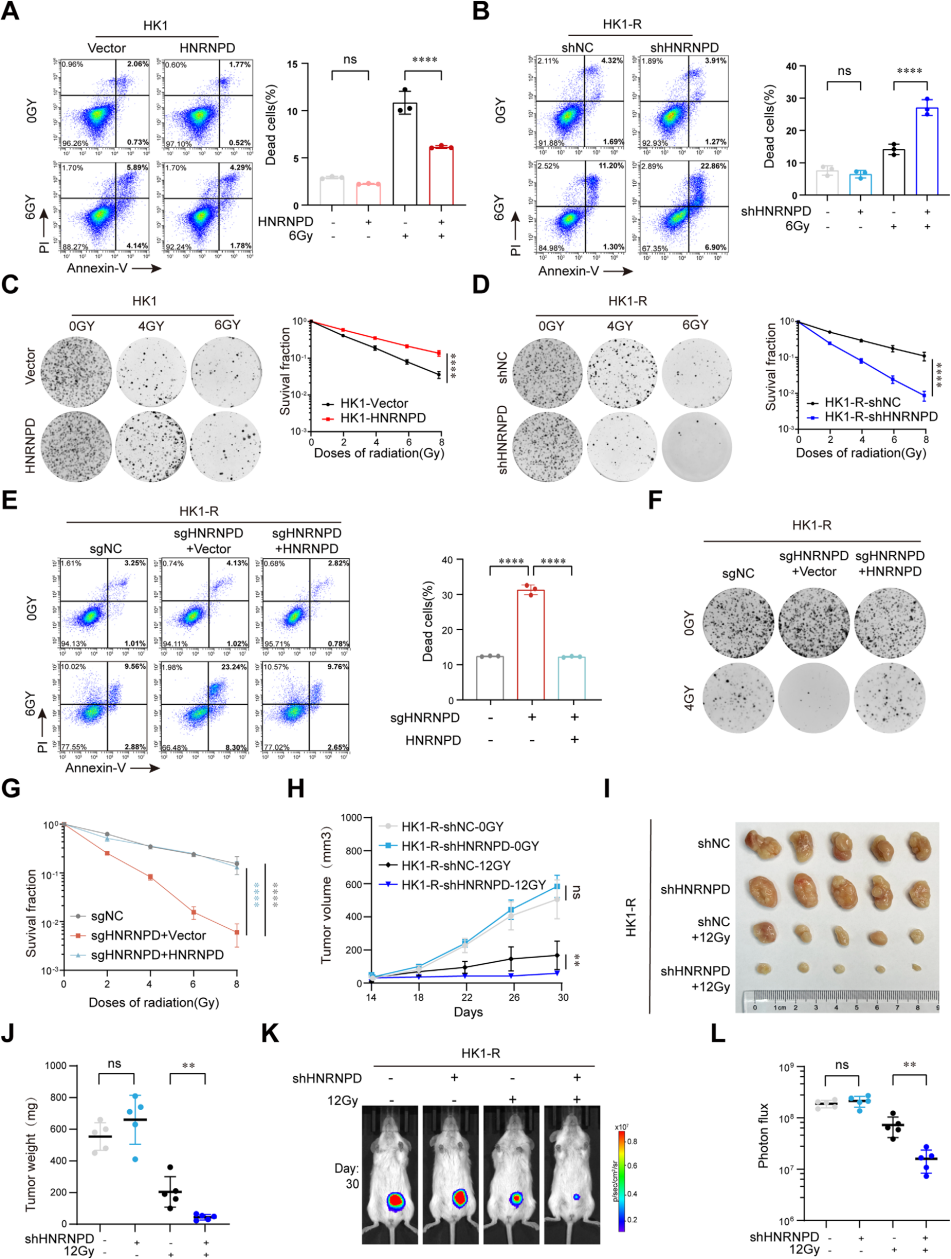
HNRNPD promotes radioresistance in NPC cells in vitro and in vivo. (A, B) Left, representative flow cytometry plots of cell death. Right, quantified percentages of dead cells in HK1‐Vector vs HK1‐HNRNPD cells (A) and HK1‐R‐shNC vs HK1‐R‐shHNRNPD cells (B) at 48 h after treatment with or without 6 Gy irradiation. Data represent mean ± SD from three independent experiments. (C, D) Left, representative images of colony formation assays. Right, clonogenic survival curves for HK1‐Vector vs HK1‐HNRNPD cells (C) and HK1‐R‐shNC vs HK1‐R‐shHNRNPD cells (D) exposed to the indicated radiation doses. Data represent mean ± SD from three independent experiments. (E) Left, representative flow cytometry plots of cell death. Right, quantified percentages of dead cells in HK1‐R cells transduced with sgNC, sgHNRNPD + Vector, or sgHNRNPD + HNRNPD at 48 h after 6 Gy irradiation. Data represent mean ± SD from three independent experiments. (F, G) Representative colony formation images after 0 Gy or 4 Gy irradiation (F) and clonogenic survival curves at the indicated radiation doses (G) in HK1‐R cells transduced with sgNC, sgHNRNPD + Vector, or sgHNRNPD + HNRNPD. Data represent mean ± SD from three independent experiments. (H) Growth curves of HK1‐R xenograft tumors in mice bearing shNC or shHNRNPD cells with or without radiotherapy (RT, 2 Gy/fraction, 6 fractions). Data represent mean ± SD (*n* = 5 mice per group). (I) Representative images of excised tumors at the experimental endpoint (day 30). (J) Tumor weights at day 30. Data represent mean ± SD (*n* = 5 tumors per group). (K) Representative in vivo bioluminescence images of tumors from the indicated groups on day 30. (L) Quantification of total photon flux from (K). Data represent mean ± SD (*n* = 5). ns, not significant; **p < 0.01; ****p < 0.0001; statistical significance was determined by two‐sided unpaired Student's *t*‐test (A, B, J, L), one‐way ANOVA followed by Šídák's multiple‐comparisons test (E), and two‐way ANOVA followed by Šídák's multiple‐comparisons test (C, D, G, H).

Because radiation‐induced cell death is a major determinant of radiosensitivity, we next examined cell death by flow cytometry in the presence or absence of irradiation. Under non‐irradiated conditions, no significant differences were observed among the indicated groups. However, after 6 Gy irradiation, HNRNPD overexpression significantly reduced radiation‐induced cell death in HK1 and C666‐1 cells, whereas HNRNPD knockdown markedly increased cell death in HK1‐R and C666‐1‐R cells (Figure 3A,B; Supplementary Figure S6E,F). Consistently, clonogenic assays showed that HNRNPD overexpression enhanced radioresistance, whereas HNRNPD depletion restored radiosensitivity in resistant cells (Figure 3C,D; Supplementary Figure S6G,H).

To further exclude potential off‐target effects and confirm the specificity of this phenotype, we performed CRISPR‐mediated knockout‐and‐rescue experiments in HK1‐R cells. Successful HNRNPD knockout and re‐expression were confirmed by immunoblotting (Supplementary Figure S6I). Functionally, HNRNPD knockout increased radiation‐induced cell death, whereas re‐expression of HNRNPD reversed this effect (Figure 3E). Similarly, HNRNPD knockout markedly reduced clonogenic survival after irradiation, and HNRNPD re‐expression restored radioresistance (Figure 3F,G).

We then assessed the effect of HNRNPD on tumor radiosensitivity in vivo. In HK1‐R xenografts, HNRNPD knockdown significantly enhanced the response to radiotherapy, leading to slower tumor growth, smaller tumors, lower tumor weights, and weaker bioluminescence signals compared with control tumors after irradiation (Figure 3H–L). Conversely, in a complementary C666‐1 xenograft model, HNRNPD overexpression attenuated the antitumor effect of radiotherapy, resulting in accelerated tumor growth, increased tumor burden, and stronger bioluminescence signals after irradiation (Supplementary Figure S6J–N).

Collectively, these findings demonstrate that HNRNPD is a key promoter of NPC radioresistance, whereas its depletion enhances radiation‐induced cell death and sensitizes tumors to radiotherapy.

### 2.4 HNRNPD Forms Biomolecular Condensates via Its C‐terminal IDR

**FIGURE 4:**
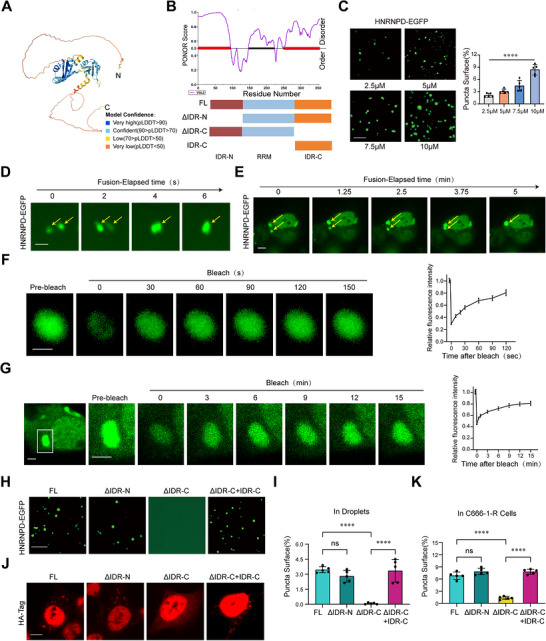
HNRNPD forms biomolecular condensates via its C‐terminal intrinsically disordered region (IDR). (A) Predicted structure of HNRNPD by AlphaFold (*Source*: UniProt). N and C termini are indicated. (B) Top, intrinsic disorder propensity of HNRNPD predicted by the PONDR VSL2 algorithm (score > 0.5 indicates disorder). Bottom, schematic of full‐length (FL) and truncated HNRNPD constructs (ΔIDR‐N, ΔIDR‐C, IDR‐C). (C) Top, representative images of in vitro droplet formation by recombinant HNRNPD‐EGFP‐3HA protein at the indicated concentrations in the presence of 12.5 ng/µL total RNA and 10% PEG‐8000. Scale bar, 5 µm. Bottom, quantification of droplet area from five random fields. (D) Time‐lapse images showing fusion events of HNRNPD‐EGFP‐3HA droplets in vitro. Scale bar, 2 µm. (E) Time‐lapse images showing fusion behavior of HNRNPD‐EGFP condensates in live C666‐1‐R cells after 6 Gy irradiation. Scale bar, 5 µm. (F) Left, representative images from a fluorescence recovery after photobleaching (FRAP) assay performed on in vitro HNRNPD‐EGFP‐3HA droplets. Scale bar, 1 µm. Right, quantification of fluorescence recovery over time (*n* = 3 droplets). (G) Left, representative FRAP images of HNRNPD‐EGFP condensates in live C666‐1‐R cells after irradiation. Scale bar, 2 µm. Right, quantification of fluorescence recovery (*n* = 3 cells). (H) Representative images of in vitro droplet formation by the indicated purified HNRNPD proteins (10 µM) with 12.5 ng/µL total RNA and 10% PEG‐8000. Scale bar, 5 µm. (I) Quantification of droplet area from five random fields for each condition in (H). (J) Representative confocal microscopy images of C666‐1‐R cells transfected with the indicated HNRNPD‐3HA constructs, irradiated (6 Gy), and stained with anti‐HA antibody [30] to visualize condensates. Scale bar, 5 µm. (K) Quantification of the cytoplasmic area occupied by HA‐positive puncta from (J). Data in C, I, K represent mean ± SD of five independent experiments. ns, not significant; ****p < 0.0001; statistical significance was determined by two‐sided unpaired Student's *t*‐test (C) and one‐way ANOVA followed by Šídák's multiple‐comparisons test (I, K).

Many RBPs within SGs contain intrinsically disordered regions (IDRs) that promote liquid–liquid phase separation (LLPS) and condensate formation [19]. HNRNPD is an RBP with two RNA‐binding motifs. To determine whether HNRNPD has LLPS properties, we analyzed its amino acid sequence using AlphaFold structure prediction (Figure 4A) and VSL2 disorder prediction models (Figure 4B). The results revealed two IDRs in HNRNPD, one at the N‐terminus (aa 1–97) and one at the C‐terminus (aa 258–355), suggesting that HNRNPD may undergo LLPS through these structural domains.

Next, we purified the recombinant HNRNPD–EGFP–3HA fusion protein (Supplementary Figure S7A) and observed that it formed spherical droplets in the presence of 12.5 ng/µL total RNA extract and 10% PEG‐8000, with the droplet size proportional to the protein concentration (Figure 4C). Treatment with 1,6‐hexanediol (1,6‐HD) dissolved HNRNPD‐EGFP‐3HA droplets in vitro (Supplementary Figure S7B). Furthermore, the HNRNPD‐EGFP‐3HA aggregates exhibited both fusion (Figure 4D) and fission behaviors (Supplementary Figure S7C). Fluorescence recovery after photobleaching (FRAP) revealed rapid recovery of HNRNPD‐EGFP‐3HA droplets (Figure 4F), a characteristic of phase‐separated condensates. Moreover, in C666‐1‐R cells overexpressing EGFP‐tagged HNRNPD, we observed fusion behavior (Figure 4E), fission behavior (Supplementary Figure S7D), and stable signal recovery in the FRAP (Figure 4G) of HNRNPD‐EGFP cytoplasmic aggregates following 6 Gy of radiation exposure, mirroring the behavior of HNRNPD‐EGFP droplets.

To determine which IDR is needed, we generated ΔIDR‐N and ΔIDR‐C constructs [20]. Deletion of the C‐terminal IDR markedly impeded droplet formation, whereas reintroduction of the C‐terminal IDR restored both in vitro and intracellular condensate formation (Figure 4H–K). Given that glycine–tyrosine‐rich motifs are known to drive LLPS in RBPs and that such a motif is present in the C‐terminus of HNRNPD, our data suggest that the C‐terminal IDR of HNRNPD is the key domain mediating its phase separation [21, 22]. Together, these data demonstrate that HNRNPD forms biomolecular condensates through its C‐terminal IDR.

### 2.5 Irradiation Promotes Cytoplasmic Accumulation of HNRNPD, and the p37 Isoform Links HNRNPD to G3BP1‐Dependent SG Assembly

**FIGURE 5:**
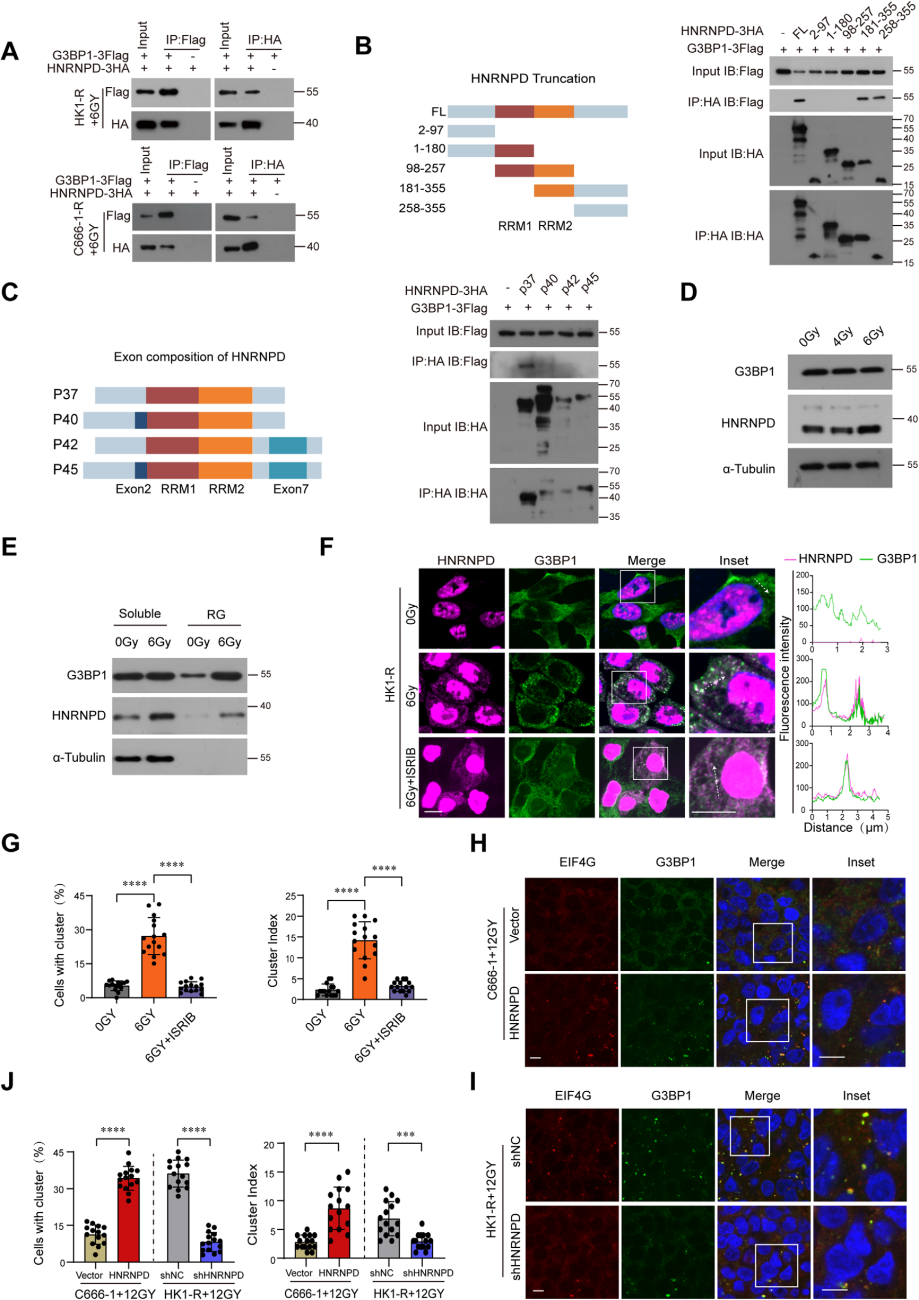
Irradiation promotes cytoplasmic accumulation of HNRNPD, and the p37 isoform links HNRNPD to G3BP1‐dependent SG assembly. (A) Co‐immunoprecipitation (Co‐IP) assays using cell lysates from NPC cells co‐transfected with G3BP1‐3Flag and HNRNPD‐3HA with 6 Gy irradiation. (B) Left, schematic of HNRNPD truncation mutants. Right, Co‐IP (using anti‐HA beads) and immunoblot analysis of lysates from HK1‐R cells co‐transfected with G3BP1‐3Flag and the indicated HNRNPD‐3HA truncations. (C) Left, schematic of the four major HNRNPD isoforms (p37, p40, p42, p45). Right, Co‐IP and immunoblot analysis of lysates from HK1‐R cells co‐transfected with G3BP1‐3Flag and the indicated HNRNPD‐3HA isoforms. (D) Western blot analysis of whole‐cell lysates from HK1‐R cells after the indicated doses of irradiation. (E) Following subcellular fractionation with or without radiation exposure, soluble cytoplasmic lysates and insoluble RNP granules (RG) containing SGs were analyzed by Western blot. (F) Representative immunofluorescence images showing the localization of endogenous HNRNPD (magenta) and G3BP1 in HK1‐R cells under the indicated conditions. Scale bar, 5 µm. Insets show magnified views. Inset scale bar, 5 µm. (G) Quantification of the percentage of cells with SGs and the SG cluster index from (F). Data are expressed as mean ± SD (*n* = 15 image fields per condition from three independent experiments). (H, I) Representative confocal IF images of G3BP1, eIF4G, and DAPI (blue) in sections from the indicated xenograft tumors. Scale bar, 5 µm. Inset scale bar, 5 µm. (J) Quantification of the percentage of SG‐positive cells and the SG cluster index from (H, I). Data are presented as mean ± SD (*n* = 15 image fields per condition from three independent tumors). ***p < 0.001, ****p < 0.0001; statistical significance was determined by one‐way ANOVA followed by Šídák's multiple‐comparisons test (G) and two‐sided unpaired Student's *t*‐test (J).

SGs are membrane‐less organelles formed by cells under stress conditions, and are composed mainly of translation‐arrested messenger ribonucleoprotein (mRNP) complexes [21, 22]. During stress, an increase in mRNP concentration triggers a conformational shift of the IDR of G3BP1 from an autoinhibitory state to an RNA‐binding dimer, which serves as a molecular switch for SG nucleation [23, 24]. Given our finding that HNRNPD undergoes RNA‐dependent phase separation, we next evaluated its interaction with G3BP1. Coimmunoprecipitation (Co‐IP) revealed that HNRNPD interacted with G3BP1 after irradiation (Figure 5A). Truncation analysis revealed that only the construct containing the C‐terminal IDR (aa 258–355) could bind to G3BP1 (Figure 5B).

Alternative splicing generates four HNRNPD isoforms, p37, p40, p42, and p45 [25]. Co‐IP analysis of these isoforms showed that only p37 clearly interacted with G3BP1, whereas p40, p42, and p45 did not show detectable binding (Figure 5C). To further clarify the roles of distinct HNRNPD isoforms in the radiation response of NPC, we compared their expression characteristics and functional effects. Untagged p37, p40, p42, and p45 were individually re‐expressed in HNRNPD‐knockout HK1‐R cells, and their electrophoretic mobility was compared with endogenous HNRNPD bands in untreated HK1‐R cells. Immunoblot analysis showed that the two major endogenous HNRNPD bands comigrated with p37 and p45, whereas bands corresponding to p40 and p42 were weak or below the detection threshold, suggesting that p37 and p45 are the predominant detectable endogenous isoforms in HK1‐R cells (Supplementary Figure S8A). In the same reconstitution system, all four untagged isoforms were successfully expressed. Functionally, compared with the empty‐vector control, p37 re‐expression most effectively suppressed radiation‐induced cell death and restored clonogenic survival, whereas p40, p42, and p45 exhibited weaker rescue effects (Supplementary Figure S8B–D). Notably, p37 was not the most abundant re‐expressed isoform, indicating that its functional dominance cannot be simply attributed to higher protein abundance. Together, these results identify p37 as the isoform most likely to link HNRNPD to G3BP1‐dependent SG assembly and SG‐associated radioresistance in NPC cells.

We next asked whether irradiation alters HNRNPD localization. Although whole‐cell lysate analysis showed that the total protein levels of HNRNPD and G3BP1 remained largely unchanged after irradiation (Figure 5D), irradiation clearly promoted cytoplasmic accumulation of HNRNPD. In cytoplasmic fractions, HNRNPD levels increased after 6 Gy irradiation, whereas treatment with the nuclear export inhibitor leptomycin B (LMB) attenuated this increase; conversely, in nuclear fractions, irradiation reduced HNRNPD abundance, whereas LMB maintained its nuclear retention (Supplementary Figure S8E,F). Consistent with this, Co‐IP analysis showed that irradiation enhanced the interaction between HNRNPD and the nuclear export receptor XPO1, and this interaction was weakened by LMB treatment (Supplementary Figure S8G), indicating that radiation‐induced HNRNPD export is at least partly XPO1 dependent. Subcellular fractionation further showed that irradiation increased HNRNPD in the insoluble RNP granule fraction together with G3BP1, consistent with enhanced recruitment into SG‐containing fractions (Figure 5E).

Immunofluorescence analysis confirmed that HNRNPD underwent cytoplasmic translocation and colocalized with G3BP1 after irradiation, whereas ISRIB largely prevented HNRNPD cytoplasmic aggregation and SG formation (Figure 5F, G; Supplementary Figure S8H,I). Consistently, additional immunoblot analysis showed that ISRIB treatment or G3BP1 knockdown did not markedly alter total HNRNPD protein levels (Supplementary Figure S8J). However, immunofluorescence and quantitative analysis revealed that ISRIB reduced the cytoplasmic accumulation of HNRNPD, whereas G3BP1 knockdown did not significantly impair HNRNPD nuclear export but attenuated its cytoplasmic punctate aggregation (Supplementary Figure S8K,L). In vivo, radiotherapy increased SG assembly in HNRNPD‐overexpressing tumors, whereas HNRNPD knockdown reduced SG formation, as indicated by eIF4G/G3BP1 co‐staining and quantitative analysis of SG‐positive cells and cluster index (Figure 5H–J).

Collectively, these findings show that irradiation promotes cytoplasmic accumulation of HNRNPD and that the p37 isoform, through its specific interaction with G3BP1, plays a dominant role in driving SG assembly in NPC cells.

### 2.6 HNRNPD Suppresses GRAMD4‐Dependent Apoptosis by Binding and Sequestering GRAMD4 mRNA via Its RRM1 Domain

**FIGURE 6:**
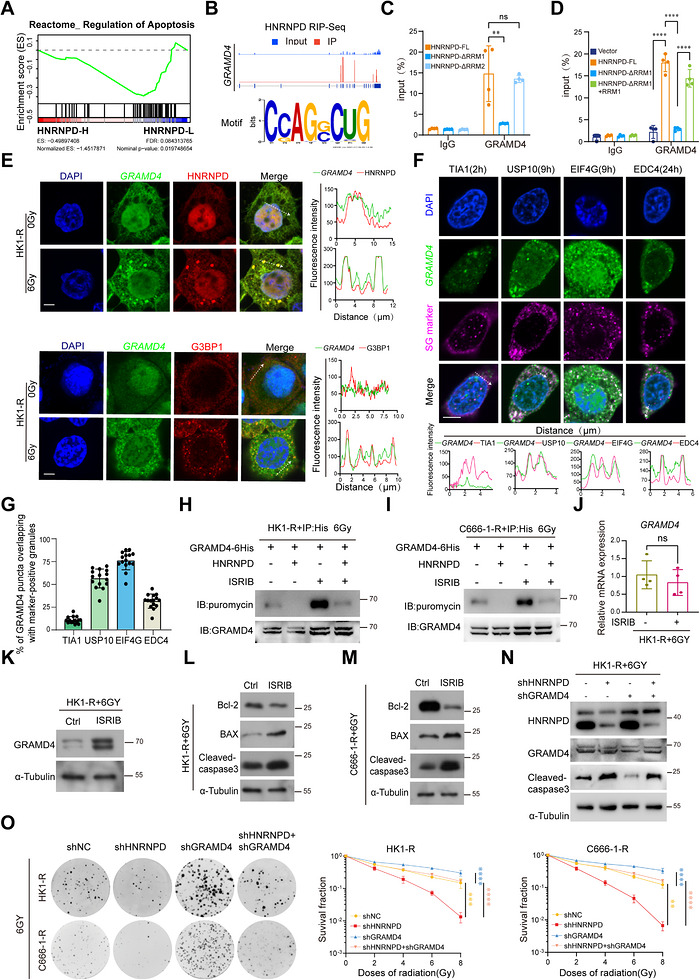
HNRNPD binds and sequesters *GRAMD4* mRNA to suppress apoptosis. (A) Gene Set Enrichment Analysis (GSEA) plot showing downregulation of the apoptosis‐related gene set “REACTOME_REGULATION_OF_APOPTOSIS” in HNRNPD‐overexpressing NPC cells after irradiation. (B) Top, Representative snapshot of HNRNPD RIP‐seq reads mapping to the *GRAMD4* mRNA locus in irradiated HK1‐R cells. Bottom, the motif enriched in HNRNPD RIP‐seq peaks identified by MEME. (C) RIP‐qPCR analysis of *GRAMD4* mRNA enrichment in anti‐HA immunoprecipitates from irradiated NPC cells expressing HNRNPD‐FL, HNRNPD‐ΔRRM1, or HNRNPD‐ΔRRM2. IgG was used as a control. Data represent mean ± SD (*n* = 4). (D) RIP‐qPCR analysis of *GRAMD4* mRNA enrichment in anti‐HA immunoprecipitates from irradiated NPC cells expressing vector, HNRNPD‐FL, HNRNPD‐ΔRRM1, or HNRNPD‐ΔRRM1 + RRM1. Data represent mean ± SD (*n* = 4). (E) Representative images of combined immunofluorescence for HNRNPD and RNA fluorescence in situ hybridization (FISH) for *GRAMD4* mRNA (top), and for G3BP1 and FISH for *GRAMD4* mRNA (bottom), in HK1‐R cells with or without 6 Gy irradiation. Right, fluorescence intensity line profiles along the indicated lines. Scale bar, 5 µm. (F) Representative images of combined immunofluorescence for the indicated stress granule markers and FISH for *GRAMD4* mRNA in irradiated HK1‐R cells. TIA1 was examined at 2 h after irradiation, USP10 and eIF4G at 9 h, and EDC4 at 24 h. Bottom, fluorescence intensity line profiles. (G) Quantification of the percentage of GRAMD4 puncta overlapping with granules positive for the indicated stress granule markers. Data represent mean ± SD (*n* = 15 image fields per condition from three independent experiments). (H, I) Analysis of nascent GRAMD4 translation in HK1‐R (H) and C666‐1‐R (I) cells after 6 Gy irradiation by anti‐His immunoprecipitation of GRAMD4‐6His followed by immunoblotting for puromycin and GRAMD4 in the presence or absence of HNRNPD and ISRIB. (J) qRT‐PCR analysis of *GRAMD4* mRNA levels in HK1‐R cells treated with or without 2 µM ISRIB for 4 h after 6 Gy irradiation. Data represent mean ± SD (*n* = 4 biological replicates). (K) Western blot analysis of GRAMD4 protein levels in HK1‐R cells treated with or without ISRIB after 6 Gy irradiation. (L, M) Western blot analysis of the indicated apoptosis‐related proteins in HK1‐R (L) and C666‐1‐R (M) cells treated with or without ISRIB after 6 Gy irradiation. (N) Western blot analysis of HNRNPD, GRAMD4, and cleaved caspase‐3 in HK1‐R cells transduced with the indicated shRNAs after 6 Gy irradiation. (O) Left, representative colony formation images of HK1‐R and C666‐1‐R cells transduced with the indicated shRNAs after 6 Gy irradiation. Right, clonogenic survival curves after exposure to the indicated radiation doses. Data represent mean ± SD (*n* = 3 independent experiments). ns, not significant; **p < 0.01; ***p < 0.001; ****p < 0.0001; statistical significance was determined by one‐way ANOVA followed by Dunnett's multiple‐comparisons test (C), one‐way ANOVA followed by Šídák's multiple‐comparisons test (D), two‐sided unpaired Student's *t*‐test (J), and two‐way ANOVA followed by Šídák's multiple‐comparisons test (O).

Stress granules are platforms for translational repression, which is achieved by sequestering mRNAs in these granules [26]. Given the role of HNRNPD in RNA binding, we hypothesized that it might increase radioresistance by sequestering pro‐apoptotic transcripts in SGs. Transcriptome sequencing of HNRNPD‐overexpressing NPC cells after irradiation revealed downregulation of apoptosis‐related pathways according to Gene Set Enrichment Analysis (GSEA) (Figure 6A). Next, we performed RNA immunoprecipitation sequencing (RIP‐seq) in NPC cells transfected with 3HA‐labeled HNRNPD after radiotherapy. The results revealed that *GRAMD4* mRNA was enriched in the “CCAGSCUG” motif for enhanced HNRNPD binding after radiotherapy (Figure 6B). GRAMD4 is a known mediator of p73‐dependent mitochondrial apoptosis via Bax activation [27]. RIP‐qPCR verified the binding of *GRAMD4* mRNA to HNRNPD after irradiation. In addition, as HNRNPD has two RRM domains, RRM1 and RRM2, we next performed domain‐mapping analysis and demonstrated by RIP‐qPCR that the RRM1 domain is required for binding to GRAMD4 (Figure 6C). To further validate this finding, we conducted an RRM1 domain complementation assay. Immunoblot analysis confirmed the expression of full‐length HNRNPD, HNRNPD‐ΔRRM1, and the RRM1‐complemented construct used in these assays (Supplementary Figure S9A), and restoration of the RRM1 domain rescued the binding of HNRNPD to GRAMD4 mRNA (Figure 6D).

Combined RNA FISH and immunofluorescence demonstrated colocalization of *GRAMD4* mRNA with HNRNPD and with the SG marker G3BP1 in irradiated HK1‐R cells (Figure 6E). To further define the granule context of *GRAMD4* sequestration, we examined additional SG markers across different stages of SG maturation. *GRAMD4* mRNA puncta colocalized with TIA1 at 2 h after irradiation, with USP10 and eIF4G at 9 h, and with EDC4 at 24 h, indicating that *GRAMD4* mRNA is recruited into SG‐associated compartments over time (Figure 6F,G).

We next asked whether HNRNPD suppresses GRAMD4 expression at the translational level. Nascent translation assays in HK1‐R and C666‐1‐R cells showed that HNRNPD reduced newly synthesized GRAMD4 protein after irradiation, whereas ISRIB restored GRAMD4 translation in both cell lines (Figure 6H,I). Consistent with this, ISRIB did not significantly alter *GRAMD4* mRNA abundance in either HK1‐R or C666‐1‐R cells (Figure 6J; Supplementary Figure S9B), but markedly increased GRAMD4 protein expression (Figure 6K; Supplementary Figure S9C), supporting a mechanism of translational repression rather than mRNA degradation. To exclude the possibility that HNRNPD regulates GRAMD4 through proteasomal turnover, we performed ubiquitination assays in the presence or absence of MG132. Although MG132 increased the accumulation of ubiquitinated GRAMD4, HNRNPD expression did not measurably alter GRAMD4 ubiquitination, indicating that HNRNPD does not regulate GRAMD4 primarily through ubiquitin‐mediated degradation (Supplementary Figure S9D).

Functionally, ISRIB treatment enhanced radiation‐induced cell death in both HK1‐R and C666‐1‐R cells (Supplementary Figure S9E), accompanied by decreased Bcl‐2 and increased BAX and cleaved caspase‐3 levels (Figure 6L, M), indicating reactivation of the mitochondrial apoptotic pathway.

To further test whether GRAMD4 is required for the proapoptotic phenotype caused by HNRNPD depletion, we performed knockdown experiments in radioresistant NPC cells. HNRNPD knockdown increased GRAMD4 expression and cleaved caspase‐3 levels after irradiation (Figure 6N), reduced clonogenic survival (Figure 6O), and increased radiation‐induced cell death; importantly, simultaneous GRAMD4 knockdown partially reversed the increase in cell death caused by HNRNPD depletion in both HK1‐R and C666‐1‐R cells (Supplementary Figure S9F,G).

Together, these findings indicate that HNRNPD binds *GRAMD4* mRNA through its RRM1 domain, promotes its sequestration in SG‐associated compartments, represses GRAMD4 translation rather than enhancing its degradation, and thereby suppresses mitochondrial apoptosis in radioresistant NPC cells.

### 2.7 HNRNPD Promotes Radioresistance by Suppressing GRAMD4‐Dependent Apoptosis In Vitro and In Vivo

**FIGURE 7:**
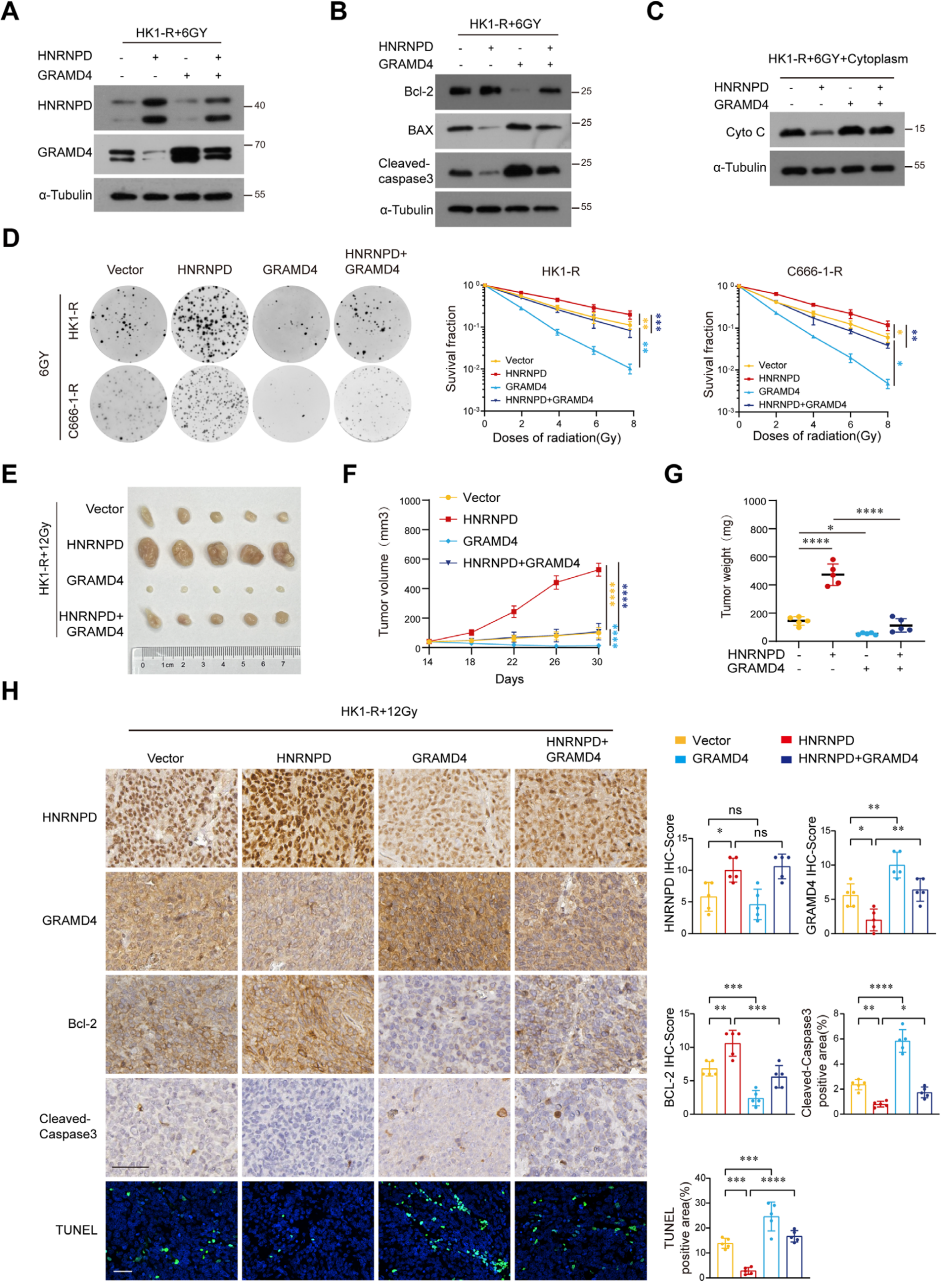
HNRNPD suppresses GRAMD4‐dependent apoptosis and promotes radioresistance in vitro and in vivo. (A) Western blot analysis confirming HNRNPD and GRAMD4 protein expression in HK1‐R cells stably transduced with the indicated constructs after 6 Gy irradiation. (B) Western blot analysis of Bcl‐2, BAX, and cleaved caspase‐3 levels in HK1‐R cells stably transduced with the indicated constructs after 6 Gy irradiation. (C) Western blot analysis of cytochrome c (Cyto C) release in the cytosolic fraction of HK1‐R cells stably transduced with the indicated constructs after 6 Gy irradiation. (D) Left, representative colony formation images of HK1‐R and C666‐1‐R cells stably transduced with the indicated constructs after 6 Gy irradiation. Right, clonogenic survival curves after exposure to the indicated radiation doses. Data represent mean ± SD (*n* = 3 independent experiments). (E) Representative images of excised tumors from HK1‐R xenografts at the experimental endpoint after radiotherapy (total dose, 12 Gy). (F) Tumor growth curves of HK1‐R xenografts in mice receiving the indicated treatments. Data represent mean ± SD (*n* = 5 mice per group). (G) Tumor weights of HK1‐R xenografts at the experimental endpoint. Data represent mean ± SD (*n* = 5 tumors per group). (H) Left, representative images of tumor sections from the indicated groups stained for HNRNPD, GRAMD4, Bcl‐2, cleaved caspase‐3, and TUNEL. Right, quantification of HNRNPD and GRAMD4 IHC scores, Bcl‐2 IHC scores, and the percentages of cleaved caspase‐3‐positive and TUNEL‐positive areas. Data represent mean ± SD (*n* = 5 tumors per group). Scale bar, 50 µm. ns, not significant; *p < 0.05; **p < 0.01; ***p < 0.001; ****p < 0.0001; statistical significance was determined by two‐way ANOVA followed by Šídák's multiple‐comparisons test (D, F) and one‐way ANOVA followed by Šídák's multiple‐comparisons test (G, H).

To determine whether GRAMD4 mediates HNRNPD‐driven radioresistance in NPC, we restored GRAMD4 expression in radioresistant NPC cells with or without HNRNPD overexpression. Immunoblotting confirmed the expression of HNRNPD and GRAMD4 in HK1‐R cells after irradiation (Figure 7A), and parallel validation was performed in C666‐1‐R cells (Supplementary Figure S10A). Restoration of GRAMD4 reversed the antiapoptotic effects of HNRNPD, as evidenced by reduced Bcl‐2 levels, increased BAX and cleaved caspase‐3, and restored cytochrome c release after irradiation in HK1‐R cells (Figure 7B,C). Consistent changes were also observed in C666‐1‐R cells (Supplementary Figure S10B,C). Functionally, colony formation assays showed that GRAMD4 restoration markedly abrogated HNRNPD‐induced radioresistance in both HK1‐R and C666‐1‐R cells (Figure 7D). In addition, flow cytometry confirmed that GRAMD4 re‐expression reversed the suppressive effect of HNRNPD on radiation‐induced cell death in both cell lines (Supplementary Figure S10D). These findings identify GRAMD4 as a functional downstream effector of HNRNPD in regulating apoptotic responses to irradiation.

To further validate this mechanism in vivo, we established xenograft models using HK1‐R cells transduced with GRAMD4, with or without HNRNPD overexpression. HNRNPD overexpression promoted tumor radioresistance, whereas GRAMD4 restoration largely abolished this effect, resulting in smaller excised tumors, slower tumor growth, and lower tumor weights after radiotherapy (Figure 7E–G). Histological analysis further showed that GRAMD4 restoration reversed the HNRNPD‐mediated antiapoptotic phenotype in vivo, as indicated by decreased Bcl‐2 staining and increased cleaved caspase‐3 and TUNEL positivity, while HNRNPD and GRAMD4 staining confirmed successful modulation of the indicated proteins (Figure 7H). Consistent results were obtained in the C666‐1‐R xenograft model, in which GRAMD4 re‐expression suppressed tumor growth and reduced tumor weight despite HNRNPD overexpression (Supplementary Figure S10E–G). Bioluminescence imaging further supported these findings in both HK1‐R and C666‐1‐R models (Supplementary Figure S10H,I).

**FIGURE 8:**
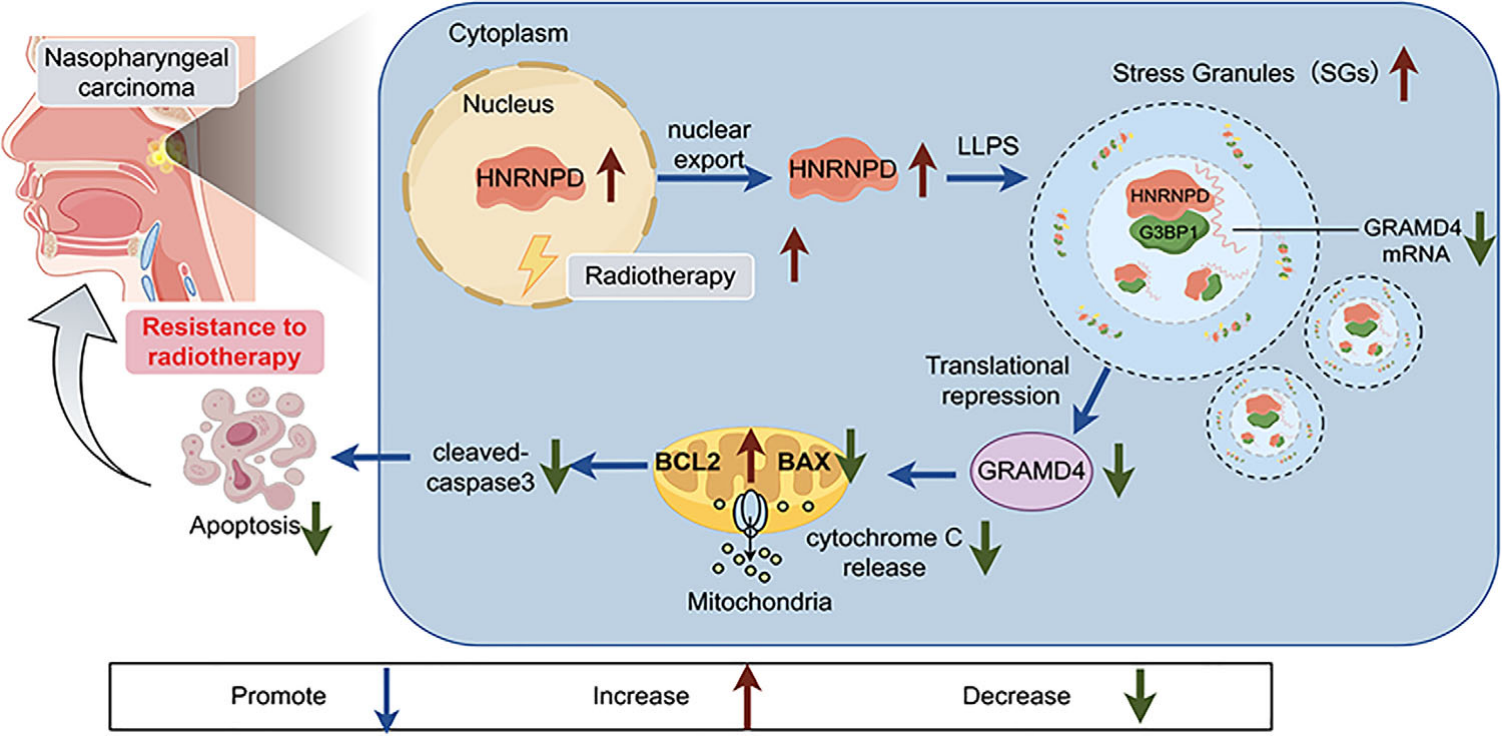
Schematic summary of the main findings in this study. Irradiation induces nuclear export and cytoplasmic accumulation of HNRNPD, which undergoes LLPS and promotes stress granule (SG) assembly. The p37 isoform specifically interacts with G3BP1 and is the functionally dominant isoform in this process. HNRNPD sequesters *GRAMD4* mRNA in SGs through its RRM1 domain, represses GRAMD4 translation, inhibits mitochondrial apoptosis, and thereby promotes radioresistance in NPC cells.

Collectively, these results demonstrate that HNRNPD enhances NPC radioresistance by suppressing GRAMD4‐dependent apoptosis, and that restoration of GRAMD4 is sufficient to counteract the radioresistant phenotype driven by HNRNPD (Figure 8).

## 3 Discussion

Stress granules (SGs) are adaptive ribonucleoprotein condensates that help cancer cells survive therapeutic stress by reorganizing untranslated mRNAs and RNA‐binding proteins (RBPs), thereby reshaping translational output and stress adaptation [22, 24]. Accumulating evidence indicates that enhanced SG formation supports therapy resistance in multiple tumor types, including RIOK1‐driven translational restriction of PTEN in hepatocellular carcinoma [28], UBAP2L‐dependent oxaliplatin resistance in gastric cancer [29], LIMK1/YTHDC2‐mediated 5‐FU chemoresistance in colorectal cancer [9], and SG‐dependent tumor adaptation in pancreatic cancer [30]. In nasopharyngeal carcinoma (NPC), SG‐related pathways have also been implicated in malignant progression and treatment response, including the DCAF7–USP10–G3BP1 axis [31]. The significance of the present study, however, is not simply that another SG‐associated regulator is involved in NPC. Rather, we define a radiotherapy‐specific and isoform‐specific posttranscriptional mechanism in which irradiation promotes HNRNPD‐dependent SG adaptation, and the p37 isoform couples this response to selective sequestration of the proapoptotic transcript GRAMD4, thereby increasing the apoptotic threshold and promoting radioresistance.

From a mechanistic perspective, our data place HNRNPD within the established framework of SG assembly while also extending it. SG formation depends on the accumulation of untranslated mRNPs, multivalent RNA–RNA and RNA–protein interactions, and intrinsically disordered region (IDR)‐driven phase separation [6, 23, 24]. G3BP1 is a core nucleator of mammalian SGs [6], and our finding that HNRNPD contains a functional C‐terminal IDR that forms RNA‐dependent condensates and binds G3BP1 supports the idea that HNRNPD actively participates in SG assembly rather than merely localizing to pre‐existing granules. More broadly, phase‐separating RBPs such as FUS and hnRNPA1 illustrate how IDR‐dependent condensation can shape granule biology and stress adaptation [13, 14]. Importantly, although isoform‐specific behavior of HNRNPD/AUF1 has been described in other systems [25, 32, 33], our study adds a distinct layer of biological specificity by showing that in NPC the p37 isoform is the functionally dominant species linking irradiation to G3BP1 interaction, SG assembly, and downstream radioresistance. Thus, the novelty here lies in connecting isoform‐selective HNRNPD behavior to a defined SG‐dependent survival mechanism under radiotherapeutic stress in NPC.

The present study also strengthens the causal interpretation between SG formation and radioresistance. Time‐course analyses showed that SG assembly is dose‐ and time‐dependent. In addition, live‐cell imaging showed that transient G3BP1‐positive granules formed in more irradiated HK1‐R cells over time than was evident from single‐frame imaging, indicating that static measurements underestimate SG engagement. Together with the results of ISRIB treatment and genetic G3BP1 depletion and rescue, these findings support SG assembly as a major adaptive arm of the radiation response in NPC. At the same time, our data do not exclude the possibility that HNRNPD also contributes to radiosensitivity through additional stress‐response pathways, but they clearly establish SG dynamics as a central component of its radioprotective function. This interpretation is also consistent with the elevated basal and irradiation‐induced eIF2α phosphorylation observed in radioresistant cells, which provides a mechanistic context for the preferential radiosensitizing effect of ISRIB [17, 34]. More generally, the functional output of SGs is known to vary with stress type, cellular context, and granule composition [21, 24], and a recent study in head and neck squamous cell carcinoma further highlighted context‐dependent differences in SG formation between radiosensitive and radioresistant settings [35].

A central mechanistic finding of this study is that HNRNPD suppresses mitochondrial apoptosis by controlling GRAMD4 at the posttranscriptional level. GRAMD4 is a well‐established p73‐associated proapoptotic effector that promotes Bax relocalization, cytochrome c release, and mitochondrial apoptotic signaling [27]. We show that HNRNPD binds *GRAMD4* mRNA through its RRM1 domain and that *GRAMD4* mRNA colocalizes not only with HNRNPD and G3BP1, but also with SG markers associated with different stages of granule maturation, including TIA1, USP10, eIF4G, and EDC4, with stronger colocalization observed in mature SGs than in early or late SG‐associated structures [36]. These observations suggest that *GRAMD4* sequestration is preferentially associated with mature SGs, although recruitment can already be detected across the temporal continuum of SG assembly and remodeling [21, 24].This temporal preference may be functionally relevant, because mature granules are more likely to sustain stable translational silencing than nascent or late remodeling structures. The idea that specific transcripts can be selectively partitioned into granule‐associated compartments is also consistent with previous studies showing regulated transcript sequestration in SG‐related systems, including QKI‐mediated shuttling of modified transcripts into stress granules [7], Ataxin‐2 granule‐dependent control of Xbp1 signaling [10], and PABPC1‐dependent stabilization of U‐rich mRNAs within stress granules [11].

Our data further clarify how HNRNPD regulates GRAMD4 protein expression. Together, these results indicate that HNRNPD lowers GRAMD4 expression mainly through SG‐associated mRNA sequestration and translational repression, rather than through regulation of GRAMD4 ubiquitination. Functionally, both the sufficiency and necessity of GRAMD4 as a key downstream effector of the HNRNPD axis are supported: GRAMD4 restoration reversed the antiapoptotic and radioresistant phenotypes caused by HNRNPD overexpression, whereas simultaneous GRAMD4 depletion attenuated the radiosensitization induced by HNRNPD loss. These data identify GRAMD4 as a key downstream effector of the HNRNPD axis. Nevertheless, given the broad RNA‐binding repertoire of AUF1/HNRNPD family proteins [33, 37, 38], it is unlikely that GRAMD4 is the only relevant target. A more balanced interpretation is that GRAMD4 is the best‐validated proapoptotic effector in the current radiotherapy context, while additional HNRNPD‐regulated transcripts may cooperate in controlling DNA repair, cell‐cycle adaptation, oxidative stress responses, or metabolic survival.

Another important implication of this work is that irradiation‐induced HNRNPD redistribution appears to involve canonical nuclear export machinery, although the full upstream trigger remains unresolved. We found that leptomycin B reduced cytoplasmic accumulation of HNRNPD after irradiation and weakened its association with XPO1/CRM1, supporting at least partial dependence on XPO1‐mediated export. This places the proposed model on firmer mechanistic footing and indicates that radiation‐induced HNRNPD translocation is actively regulated rather than passive. However, the upstream events that license this redistribution, such as phosphorylation, ubiquitination, or other stress‐induced posttranslational modifications, remain undefined. Thus, although the present study identifies XPO1‐linked export as an important step, future work should determine how irradiation modifies HNRNPD, especially p37, to favor cytoplasmic retention and SG entry. Beyond translational control of apoptosis, it is also plausible that the HNRNPD/SG axis intersects with DNA damage response (DDR) signaling [39].Our current data point most directly to SG‐dependent repression of GRAMD4 translation and attenuation of mitochondrial apoptosis, but prior studies suggest that RBPs, including hnRNP family members, can participate more directly in DNA double‐strand break signaling, end resection, homologous recombination, R‐loop homeostasis, and local chromatin‐associated responses. HNRNPD itself has been implicated in DNA damage‐associated relocalization in other systems. We therefore favor a two‐layer model: one layer, strongly supported here, is a cytoprotective SG program that reduces apoptotic competence through selective translational repression; the other, still unresolved, is a potential contribution of HNRNPD to local DDR remodeling or repair pathway choice.

Several limitations are important when considering translational relevance. Although HK1 and C666‐1 are widely used NPC models, they do not capture the full heterogeneity of NPC [2, 3]. In addition, most in vivo experiments were performed in immunodeficient NCG mice and in subcutaneous flank xenografts. These systems are suitable for defining tumor‐intrinsic mechanisms, but they do not fully model the immune contexture, mucosal microenvironment, stromal interactions, EBV‐associated biology, or anatomical features of orthotopic NPC [2, 3]. Because radiotherapy engages both tumor‐cell‐intrinsic injury responses and host antitumor immunity, our conclusions should therefore be interpreted primarily as defining a tumor‐intrinsic adaptive program. The huPBMC‐NCG model partially improves immune relevance and suggests that ISRIB does not exert its major antitumor effect simply by altering CD8+ T‐cell infiltration or Granzyme B positivity; however, this still falls short of modeling the full immune and tissue context of clinical NPC. Future validation in more clinically relevant models will be important for assessing therapeutic translatability more rigorously. Although orthotopic NPC models have been reported, and NPC patient‐derived xenograft (PDX) models are also valuable, both remain technically challenging and are not yet widely used in mechanistic radiobiology studies [40]. Future studies using orthotopic systems, NPC‐PDX models, or more advanced humanized platforms will therefore be valuable for validating the HNRNPD–SG–GRAMD4 axis in settings that better reflect the native tumor microenvironment and host context.

The translational implications of this work are nevertheless notable. ISRIB acts downstream of phosphorylated eIF2α to antagonize the integrated stress response [17], but it is not a granule‐dissolving reagent in a narrow physical sense and may have broader consequences for stress‐responsive translation and cell‐state regulation [17, 41]. Thus, while the present results strongly support ISR‐dependent SG inhibition as a major driver of its radiosensitizing effect, contributions from other ISR‐regulated outputs cannot be fully excluded. Encouragingly, in vivo analyses showed no overt systemic toxicity by body weight, organ histology, or hematologic and biochemical readouts. Conceptually, these findings suggest that transient, treatment‐timed interference with SG adaptation during radiotherapy may be more feasible than prolonged global SG suppression. More selective approaches, such as targeting the HNRNPD–G3BP1 interaction interface, blocking p37‐associated nuclear export or cytoplasmic retention, or preventing selective recruitment of proapoptotic transcripts into SGs, may ultimately provide a wider therapeutic window. In this regard, the broader physiological importance of RNA granules in normal tissues, especially in the nervous system, should also be kept in mind when considering systemic SG‐directed strategies [42].

In summary, our study defines a mechanistically coherent axis in which irradiation promotes cytoplasmic accumulation of HNRNPD, the p37 isoform preferentially engages G3BP1 to support SG assembly, and HNRNPD suppresses GRAMD4‐dependent mitochondrial apoptosis by binding and sequestering *GRAMD4* mRNA in SG‐associated compartments. In doing so, this work extends the study of radioresistance in NPC beyond classical DNA repair‐centered models toward isoform‐specific RNA fate control and translational reprogramming. Although additional work is needed to delineate upstream regulatory events, possible DDR crosstalk, and behavior in more clinically faithful models, the present findings define the HNRNPD–SG–GRAMD4 axis as an SG‐associated posttranscriptional mechanism contributing to NPC radioresistance and a potentially actionable target for radiosensitization.

## 4 Experimental Section

### 4.1 Clinical Specimens

Survival analysis was performed on 164 paraffin‐embedded specimens from patients with NPC diagnosed at the Sun Yat‐sen University Cancer Center between January 2014 and January 2015. Patients with radiosensitivity were defined as those who achieved complete remission after standard radiotherapy without long‐term recurrence, whereas patients with radioresistance were defined as those who exhibited persistent disease or early recurrence despite adequate radiation therapy. Tumor staging was based on the ninth edition of the AJCC criteria. The detailed clinical characteristics of the samples are summarized in Supplementary Table S1. This study was approved by the Ethics Committee of Sun Yat‐sen University Cancer Center (approval no. GZR2021‐029).

### 4.2 Cell Culture

HK1 (RRID:CVCL_7084), C666‐1 (RRID:CVCL_7949), and HEK293T (RRID:CVCL_0063) cell lines were obtained from the Sun Yat‐sen University Cancer Center, validated using short tandem repeat analysis, routinely tested, and demonstrated to be negative for mycoplasma contamination. The HK1 cells were maintained in Roswell Park Memorial Institute (RPMI) 1640 medium (Invitrogen, Carlsbad, CA, USA) supplemented with 10% fetal bovine serum (FBS) (HyClone, Logan, UT, USA) and 1% penicillin/streptomycin (Invitrogen, Carlsbad, CA, USA). The C666‐1 cells and HEK293T cells were maintained in Dulbecco's modified Eagle's medium (DMEM) (Gibco, Grand Island, NY, USA) supplemented with 10% FBS. All cells were cultured in a humidified atmosphere of 5% CO_2_ at 37°C.

### 4.3 In Vitro Droplet Assays

HEK293T cells transiently transfected with the pcDNA3.1(+)–3HA–EGFP–hHNRNPD plasmid were harvested 48 h after transfection. The protein lysates were incubated with Pierce Anti‐HA Magnetic Beads (Thermo Scientific, 88836) at room temperature for 1 h. After being washed, the proteins were gently eluted with HA peptide (2 mg/mL; Beyotime, P9808) to obtain the purified protein. Purified proteins were mixed with 10% PEG‐8000 (HUAYUN, HS1038) in droplet formation buffer (12.5 ng/µL total RNA extract, 50 mmol/L Tris‐HCl (pH 7.5), and 125 mmol/L NaCl). The addition of 1,6‐hexanediol (Sigma‐Aldrich, 240117) led to the rapid dissolution of protein droplets, confirming their liquid‐like nature. The protein solution was immediately deposited to form droplets on a glass‐bottomed culture dish (NEST, 801001) and imaged using a spinning disk confocal live‐cell imaging system (CSU‐W1; Nikon). The EGFP–HNRNPD droplet surface area was quantified using ImageJ.

### 4.4 Fluorescence Recovery After Photobleaching (FRAP)

For in vitro droplets, 50% of the laser power was applied to the specified spot with a dwell time of 0.5 s, and time‐lapse images were acquired every 5 or 30 s. For FRAP experiments in living cells, EGFP‐tagged HNRNPD‐transfected HK1 cells were seeded in glass bottom dishes. After photobleaching, imaging was performed using a confocal microscope (LSM880; Carl Zeiss) to capture the region of interest (ROI) around the EGFP‐HNRNPD‐labeled droplets. Fluorescence was photobleached to 50–60% of the baseline intensity and then monitored for recovery.

### 4.5 Subcellular Fractionation

Subcellular fractionation was performed according to a previously described method [43]. HK1‐R cells were treated with or without radiation. Total cell lysates were extracted using Pierce IP Lysis Buffer (Thermo Scientific, 87787) and centrifuged at 2000 × *g* for 5 min to remove the nuclei. The supernatant was collected and centrifuged at 10,000 × *g* for 10 min to further isolate RNP particles. The supernatant served as the soluble fraction. The pellet was subsequently washed three times for 10 min each with IP lysis buffer to obtain the ribonucleoprotein granule (RG) fraction. All centrifugation steps were performed at 4 °C. Subcellular proteins were then boiled in protein loading buffer for Western blot analysis.

### 4.6 Cellular Immunofluorescence

Cells were seeded in confocal microplates (NEST, 801001) and allowed to adhere, followed by irradiation or no treatment. The cells were subsequently washed with PBS, fixed with 4% paraformaldehyde for 30 min, permeabilized with 0.2% Triton X‐100 in PBS for 10 min, and blocked with goat serum (ZSGB‐Bio, ZLI‐9056) for 1 h, all at room temperature. The cells were subsequently incubated overnight at 4 °C with primary antibodies targeting HNRNPD (1:1000; Abcam; ab61193), G3BP1 (1:250; BD Biosciences; 611126), and HA‐Tag (1:1000; Cell Signaling Technology; 3724T). Corresponding Alexa Fluor 488 (BioLegend, 405319) and 647 (Invitrogen, A21244) secondary antibodies were used for visualization. Nuclei were counterstained with DAPI (Solarbio, C0065) for 5 min and mounted with anti‐fade fluorescence mounting medium (Abcam, ab104135).

### 4.7 Paraffin Section Immunofluorescence

Following section baking, dewaxing, and rehydration, the sections were treated with EDTA antigen retrieval solution (ZSGB‐Bio, ZLI‐9069) for 10 min. Subsequent steps—including blocking, primary and secondary antibody incubation, DAPI staining, and imaging—were analogous to the procedures used for cell immunofluorescence.

### 4.8 RNA FISH and Immunofluorescence

Cells on slides were fixed in in situ hybridization fixative (Servicebio, G1113) for 20 min, followed by three washes with PBS. The target areas were circled with a marker pen and then digested with proteinase K (5 µg/mL, HUAYUN, HB080) at 37 °C. Prehybridization buffer (Servicebio, G3046) was added, and the samples were incubated at 37 °C for 1 h. After removal, a 488‐labeled *GRAMD4* mRNA probe (sequence: 5′‐GCTCGTAGAACCACTTCTGCAGGCCAAAGTTAGTGACCGGCTTGGCACGGCACGATGCTCCACTGTATCCGCGGCTGGACCCACATGAACAAGTTCCCTCCAGATGATATAGGGCGTGTC‐3′) was added and incubated overnight at 37 °C. The hybridization solution was removed by sequential washes in 2× SSC (Servicebio, G3015) at 37 °C for 10 min, 1× SSC at 37 °C for 2 × 5 min, and 0.5× SSC at 37 °C for 10 min. The sections were then incubated with 60 µL of prewarmed branch probe hybridization solution at 40 °C for 45 min in a humid chamber. After sequential washes (2×, 1×, 0.5×, and 0.1× SSC, each at 40 °C for 5 min), signal probe hybridization solution (1:200) was added and incubated at 32 °C for 3 h, followed by SSC washes as above. Subsequent blocking, antibody incubation, DAPI staining, and imaging followed procedures similar to those for cell immunofluorescence analysis. The following primary antibodies were used for immunofluorescence staining: HNRNPD (1:1000; Abcam; ab61193), G3BP1 (1:250; BD Biosciences; 611126), USP10 (D7A5) Rabbit Monoclonal Antibody (Cell Signaling Technology, 8501, 1:200), eIF4G Antibody (Cell Signaling Technology, 2498, 1:200), anti‐TIA1 antibody (Abcam, ab140595, 1:200), and anti‐EDC4 antibody (Abcam, ab72408,1:200).

### 4.9 RIP‐seq and RIP‐qPCR

RIP assays were performed using an RNA immunoprecipitation kit (Bersinbio, Bes5101, China) according to the manufacturer's instructions. Briefly, HK1‐R cells transfected with 3HA‐HNRNPD were irradiated as indicated and lysed in RIP buffer supplemented with protease inhibitors and RNase inhibitors to preserve RNA–protein complexes and minimize RNA degradation. The clarified lysates were divided into three fractions:0.8 mL for the anti‐HA immunoprecipitation group,0.8 mL for the IgG negative control group, and 0.1 mL reserved as the input control [24]. The anti‐HA and IgG samples were incubated overnight at 4 °C with 1 µg anti‐HA antibody or 1 µg control IgG antibody, respectively. Pre‐equilibrated protein A/G beads were then added and incubated at 4 °C for 1 h. After immunoprecipitation, RNA was extracted using the TRIzol–chloroform method and subjected to RIP‐seq and qRT‐PCR analyses.

### 4.10 Mouse Xenograft Models

Female NCG mice (4 weeks old) were purchased from GemPharmatech and housed under specific pathogen‐free conditions at 22 ± 2 °C with 50–60% relative humidity on a 12 h light/12 h dark cycle. Mice were provided with standard sterilized rodent chow and autoclaved water ad libitum. For conventional subcutaneous xenograft experiments, mice were randomly assigned to the indicated groups and inoculated subcutaneously with 1 × 10^6^ specified cells. Tumor size was measured with calipers, and tumor volume was calculated using the formula: volume = (length × width^2^)/2. When tumors reached 30–50 mm^3^, mice in the radiotherapy group received local irradiation at a total dose of 12 Gy (2 Gy per fraction for 6 fractions). In groups that received ISRIB (MedChemExpress, HY‐12495), mice were administered 2.5 mg/kg ISRIB by intraperitoneal injection after each radiotherapy session, whereas control mice received the corresponding vehicle [41]. Tumor size was monitored every 4 days. At the experimental endpoint, tumor burden was assessed using an IVIS Lumina II system and Living Image software (Caliper). Tumors were then excised, weighed, and fixed for paraffin embedding followed by immunohistochemical and TUNEL staining. All animal experiments were approved by the Institutional Animal Care and Use Committee of Sun Yat‐sen University (ethical approval No. L102012022030F).

### 4.11 Humanized PBMC‐NCG Subcutaneous Xenograft Model

For the humanized xenograft model, NCG mice received 1 × 10^7^ human peripheral blood mononuclear cells (PBMCs) from a single healthy donor via tail‐vein injection. Human immune cell engraftment was monitored in peripheral blood collected from the retro‐orbital sinus on days 7, 14, and 21 after PBMC transfer by flow‐cytometric analysis of hCD45. Successful humanization was predefined as hCD45^+^ cells >10% on day 14, and all 10 mice met this criterion. On day 14 after PBMC engraftment, mice were inoculated subcutaneously with 5 × 10^6^ HK1 cells. When tumors reached 30–50 mm^3^, mice were randomized to the indicated groups and treated with local radiotherapy at 2 Gy every other day for 6 fractions. ISRIB was administered at 2.5 mg/kg by intraperitoneal injection after each radiotherapy session, whereas control mice received the corresponding vehicle. Tumor growth, body weight, and general condition were monitored throughout the experiment, and tumors were harvested at the endpoint for downstream analyses.

### 4.12 Statistical Analysis

Statistical analyses were performed using GraphPad Prism 10 and SPSS Statistics 26.0. RNA‐seq data were normalized and analyzed using DESeq2 as described above. For high‐throughput datasets, including RNA‐seq, RIP‐seq, and proteomics, differential analyses were conducted using appropriate statistical models, and *p* values were adjusted for multiple testing using the Benjamini–Hochberg false discovery rate (FDR) method.

For qRT‐PCR, gene expression was normalized to GAPDH and analyzed using the 2^−ΔCt^ or 2^−ΔΔCt^ method. For colony formation assays, surviving fractions were normalized to plating efficiency. Data are presented as mean ± SD unless otherwise indicated. Sample sizes (*n*) for each experiment are provided in the corresponding figure legends; for in vitro experiments, *n* refers to independent biological replicates unless otherwise specified, whereas for in vivo experiments *n* refers to individual mice or tumors, and for clinical analyses n refers to patients. No data points were excluded unless technical failure was evident.

For comparisons between two groups, two‐sided Student's *t*‐test (paired or unpaired, as appropriate) was used. For comparisons among multiple groups, one‐way ANOVA was used, followed by post hoc multiple‐comparisons tests as appropriate, including Dunnett's test for comparisons of multiple groups against a single control and Šídák's test for pairwise comparisons. Two‐way ANOVA was used for tumor growth curves and dose–survival curves, followed by Šídák's multiple‐comparisons test where applicable.

Differences in survival between patient subgroups were assessed using Kaplan–Meier analysis with the log‐rank test. Associations between HNRNPD expression and clinicopathological variables were analyzed using the chi‐square test. Univariate and multivariate Cox proportional hazards regression models were used to identify prognostic factors. A two‐sided *p* < 0.05 was considered statistically significant. Significance levels are indicated as *p < 0.05, **p < 0.01, ***p < 0.001, and ****p < 0.0001.

## Author Contributions

Y.‐Z.L. designed the study, performed most of the experiments, and wrote the article. T.X. provided financial support, participated in some of the experiments, and helped to write the article, and Y.‐Y.L. collected pathological specimens for immunohistochemical staining and analyzed the data. J.‐J.Z. and D.‐S.W. provided technical guidance for some experiments. H.C. and Y.T. participated in the animal experiments; S.‐Z.X. helped construct the HK1‐R and C666‐1‐R cells; H.Z. helped with the immunofluorescence experiments; and J.L. and X.‐Y.Y. helped with the flow cytometry experiments. M.‐P.D. and Q.‐Z.P. were responsible for the design of the study, providing theoretical guidance and revising the article. J.‐C.X. was responsible for the design of the study, providing financial and theoretical guidance, and revising the article.

## Author Disclosures

No author disclosures were reported.

## Conflicts of Interest

The authors declare no conflicts of interest.

## Supporting information




**Supporting File 1**: advs75577‐sup‐0001‐SuppMat.docx.

**Supporting File 2**: advs75577‐sup‐0002‐VideoS1.mp4.

**Supporting File 3**: advs75577‐sup‐0003‐Data.xlsx.

## Data Availability

Normalized RNA‐seq data from public NPC datasets were obtained from The Cancer Genome Atlas (TCGA) through the UCSC Xena browser (https://xenabrowser.net/, University of California, Santa Cruz). The RNA‐seq data supporting Figure 2A and 2B have been deposited in the NCBI Sequence Read Archive (SRA) under BioProject accession number PRJNA1463130. The RIP‐seq data generated in this study have been deposited in the NCBI SRA under BioProject accession number PRJNA1331733.The uncropped images and source data underlying the figures in this study have been deposited in Mendeley Data and are publicly available at https://doi.org/10.17632/6g9yy6hkmv.1, and have also been uploaded to the submission system as supplementary materials. Additional raw data are available from the corresponding author upon reasonable request.
