# Supplementary material for: HNRNPD Induces Radioresistance in Nasopharyngeal Carcinoma by Sequestering GRAMD4 mRNA in Stress Granules
Source: Adv Sci (Weinh). 2026 May 10;13(43):e21038. doi: 10.1002/advs.202521038 (PMC13335965; doi:10.1002/advs.202521038)
Supplement: Supplementary file 1 — Supporting File 1: advs75577‐sup‐0001‐SuppMat.docx. [file ADVS-13-e21038-s003.docx]

**Supplementary Figure Legends**

**
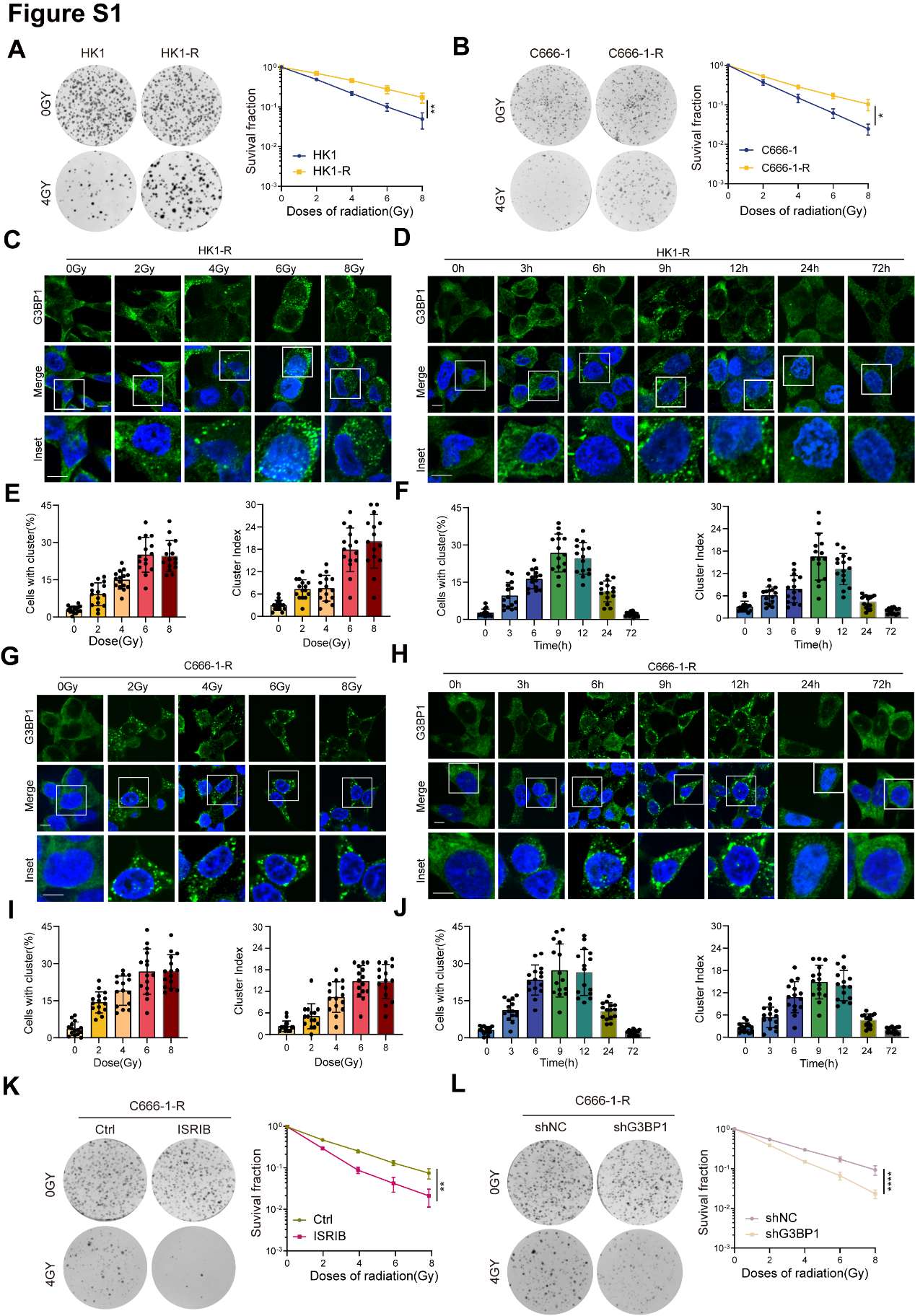
**

**Figure S1. Stress granule formation promotes radioresistance in nasopharyngeal carcinoma.**(A, B) Left, evaluation of the colony-forming ability of HK1, HK1-R, C666-1, and C666-1-R cells 10–14 days after single-dose irradiation with 0, 2, 4, 6, or 8 Gy. Right, survival fraction calculated by comparing colony formation in each treated group with that in untreated controls (0 Gy).(C, D) Representative confocal microscopy images of G3BP1 in HK1-R cells after radiation exposure, showing dose- and time-dependent changes. Nuclei were stained with DAPI (blue). Scale bar, 5 μm. Inset scale bar, 5 μm.(E, F) Quantification of the percentage of cells containing SG clusters and the SG cluster index (mean number of SGs per positive cell). Data are expressed as mean ± SD (*n* = 15 image fields per condition from three independent experiments).(G, H) Representative confocal microscopy images of G3BP1 in C666-1-R cells after radiation exposure, showing dose- and time-dependent changes. Nuclei were stained with DAPI (blue). Scale bar, 5μm. Inset scale bar, 5μm.(I, J) Quantification of the percentage of SG-positive cells and the SG cluster index. Data are presented as mean ± SD (*n* = 15 image fields per condition from three independent experiments).(K) Left, schematic of the experimental timeline for colony formation assays. Right, clonogenic survival curves for C666-1-R cells treated with or without ISRIB following exposure to the indicated doses of radiation. Data represent mean ± SD of three independent experiments.(L) Left, schematic of the experimental timeline. Right, clonogenic survival curves for C666-1-R cells transfected with control or G3BP1-targeting shRNA after exposure to the indicated radiation doses. Data represent mean ± SD of three independent experiments.**P* < 0.05, ***P* < 0.01, *****P* < 0.0001; statistical significance was determined by two-way ANOVA followed by Šídák’s multiple-comparisons test (A, B, K, L).


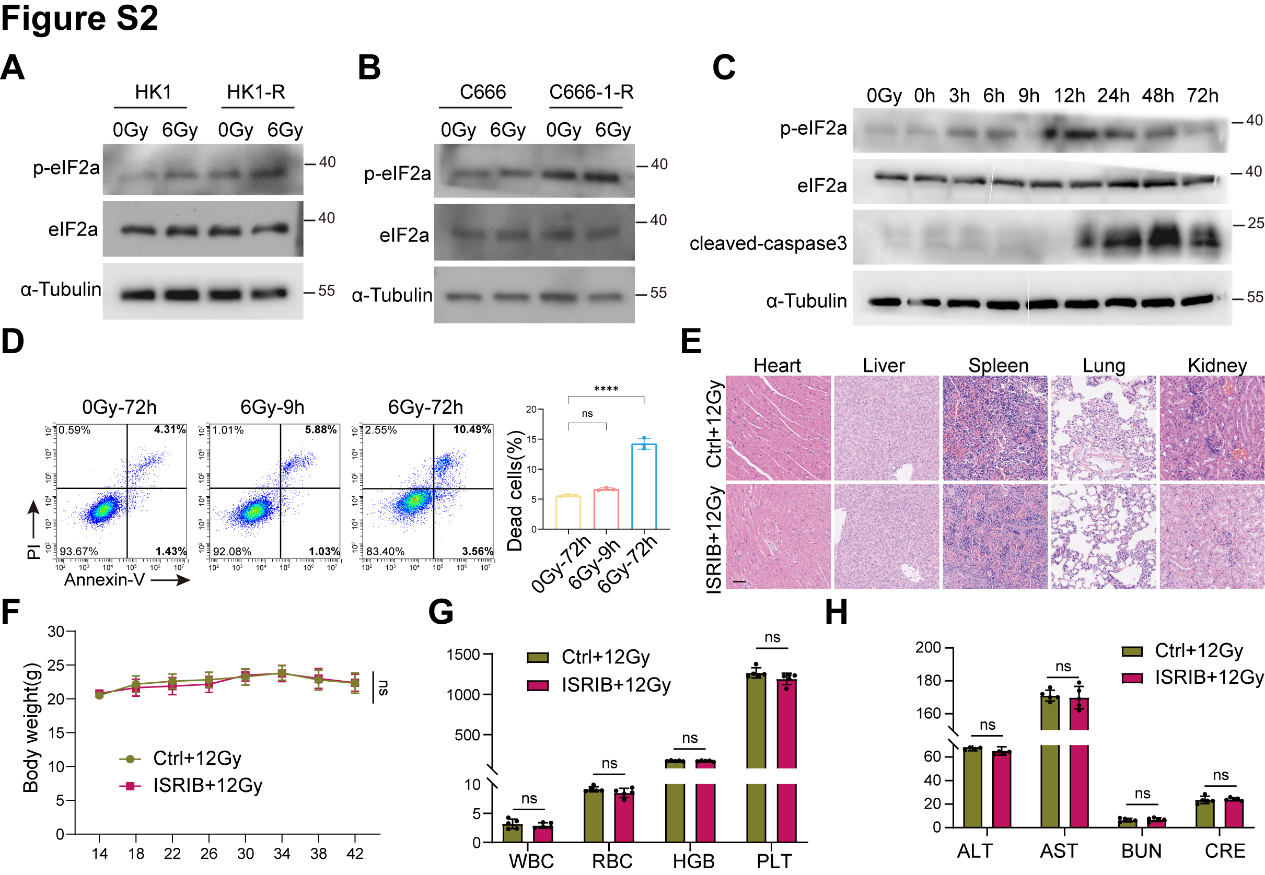


**Figure S2. p-eIF2α dynamics in radioresistant NPC cells and *in vivo* safety profile of ISRIB.(A)** Western blot analysis of p-eIF2α levels in HK1 and HK1-R cells treated with 0 or 6 Gy irradiation. Total eIF2α and α-Tubulin were used as controls.**(B)** Western blot analysis of p-eIF2α levels in C666-1 and C666-1-R cells treated with 0 or 6 Gy irradiation. Total eIF2α and α-Tubulin were used as controls.**(C)** Western blot analysis of p-eIF2α, total eIF2α, and cleaved caspase-3 in HK1-R cells collected at the indicated time points after 6 Gy irradiation. α-Tubulin was used as a loading control.**(D)** Representative flow cytometry plots of cell death and quantified percentages of dead cells in HK1-R cells under the indicated conditions (0 Gy for 72 h, 6 Gy for 9 h, and 6 Gy for 72 h). Data represent mean ± SD from three independent experiments.**（E）**Representative H&E staining images of major organs (heart, liver, spleen, lung, and kidney) from mice treated with radiotherapy alone (Ctrl+12 Gy) or radiotherapy plus ISRIB (ISRIB+12 Gy). Scale bar, 50 μm.**(F)** Body weight curves of mice in the indicated treatment groups during the experiment. Data represent mean ± SD.**(G)** Hematological parameters, including white blood cell count (WBC), red blood cell count (RBC), hemoglobin (HGB), and platelet count (PLT), in mice from the indicated treatment groups. Data represent mean ± SD.**(H)** Serum biochemical parameters, including alanine aminotransferase (ALT), aspartate aminotransferase (AST), blood urea nitrogen (BUN), and creatinine (CRE), in mice from the indicated treatment groups. Data represent mean ± SD.ns, not significant, *****P* < 0.0001; statistical significance was determined by one-way ANOVA followed by Dunnett’s multiple-comparisons test (D), two-way ANOVA followed by Šídák’s multiple-comparisons test (F), and two-sided unpaired Student’s t-test (G, H).


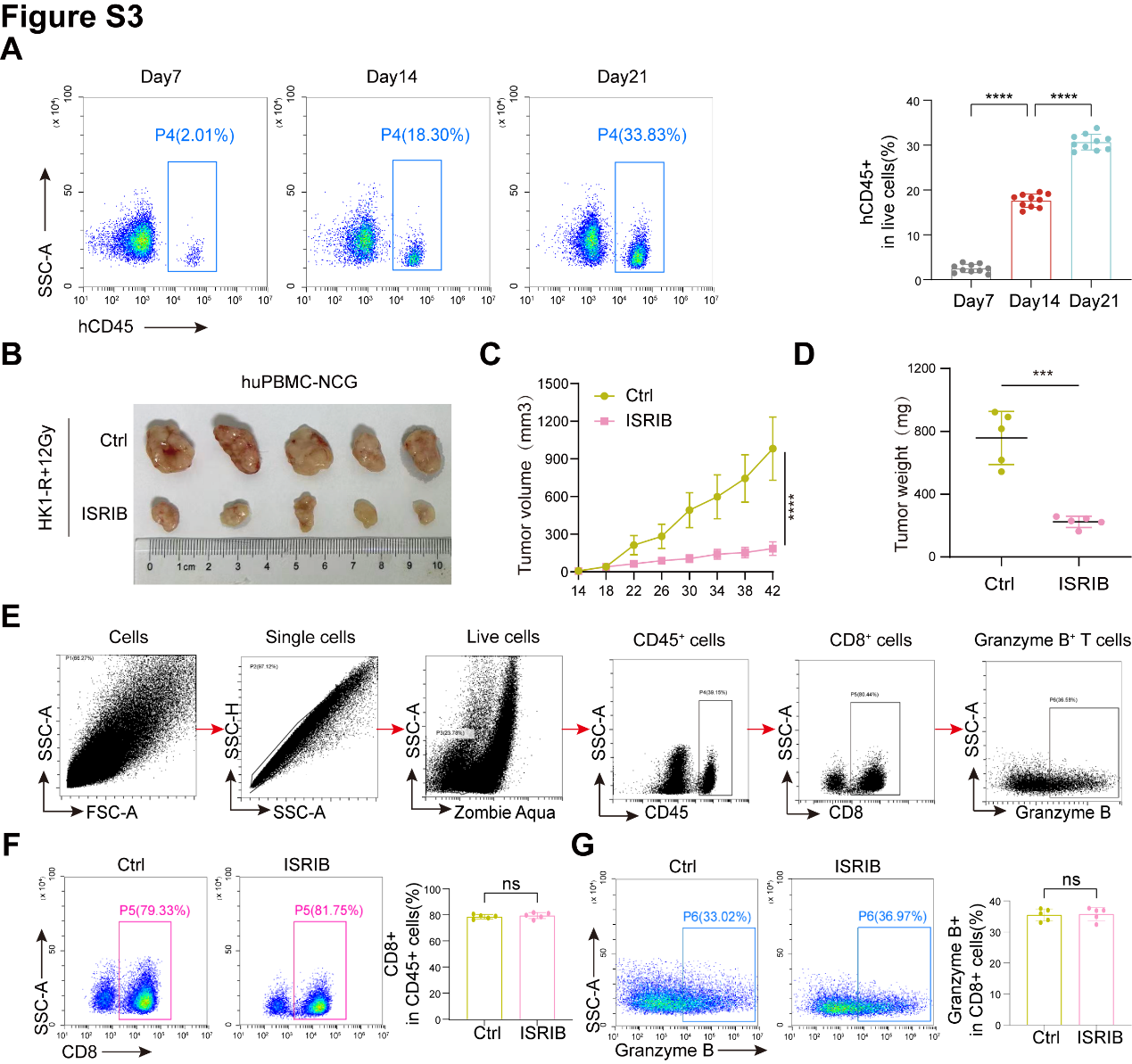


**Figure S3. ISRIB suppresses tumor growth in a humanized PBMC-NCG model.(A)** Representative flow-cytometry plots showing human immune cell engraftment in peripheral blood of huPBMC-NCG mice on days 7, 14, and 21 after PBMC transfer. Right, quantified percentages of hCD45^+^ cells in live cells at the indicated time points. Data represent mean ± SD.**(B)** Representative images of excised tumors from huPBMC-NCG mice bearing HK1-R xenografts treated with radiotherapy alone (Ctrl) or radiotherapy plus ISRIB (ISRIB) at the experimental endpoint.**(C)** Growth curves of HK1-R xenograft tumors in huPBMC-NCG mice receiving the indicated treatments. Data represent mean ± SD.**(D)** Tumor weights at the experimental endpoint in the indicated treatment groups. Data represent mean ± SD.**(E)** Representative flow-cytometry plots showing the gating strategy used for analysis of tumor-infiltrating human CD8^+^T cells and granzyme B^+^ CD8^+^ T cells in huPBMC-NCG tumors.**(F)** Left, representative flow-cytometry plots of intratumoral human CD8^+^ T cells in the indicated treatment groups. Right, quantified percentages of CD8^+^ cells among hCD45^+^ cells. Data represent mean ± SD.(G) Left, representative flow-cytometry plots of granzyme B^+^ cells within intratumoral human CD8^+^ T cells in the indicated treatment groups. Right, quantified percentages of granzyme B^+^ cells among CD8^+^ T cells. Data represent mean ± SD.ns, not significant; ****P* < 0.001; *****P* < 0.0001; statistical significance was determined by one-way ANOVA followed by Šídák’s multiple-comparisons test (A), two-way ANOVA followed by Šídák’s multiple-comparisons test (C), and two-sided unpaired Student’s *t*-test (D, F, G).


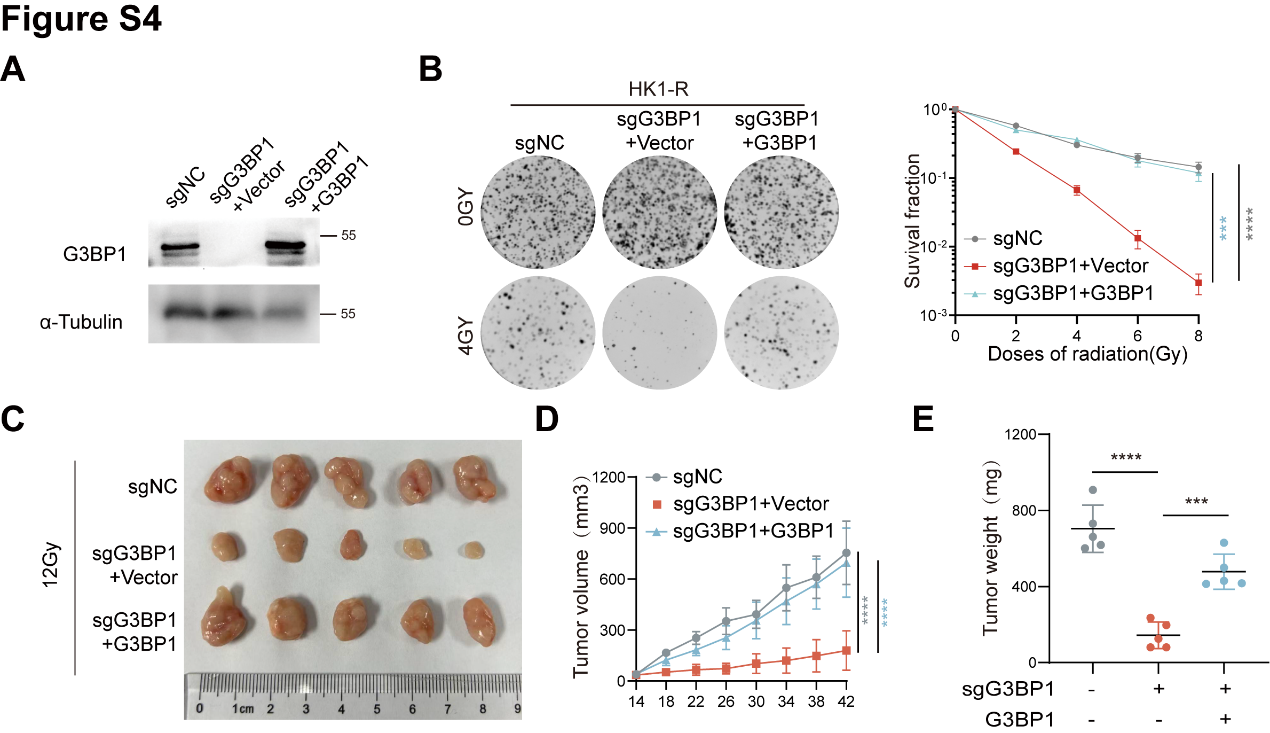


**Figure S4. G3BP1 depletion sensitizes radioresistant NPC cells to irradiation, whereas G3BP1 re-expression restores radioresistance.**(A) Immunoblot analysis confirming G3BP1 knockout and re-expression in HK1-R cells transduced with sgNC, sgG3BP1+Vector, or sgG3BP1+G3BP1. α-Tubulin served as a loading control.(B) Representative colony formation images of the indicated HK1-R cells after 0 Gy or 4 Gy irradiation (left), and corresponding clonogenic survival curves at the indicated radiation doses (right).(C) Representative images of xenograft tumors derived from the indicated HK1-R cells collected at the experimental endpoint after radiotherapy (total dose, 12 Gy).(D) Growth curves of xenografts formed by sgNC, sgG3BP1+Vector, or sgG3BP1+G3BP1 HK1-R cells following irradiation.(E) Tumor weights of xenografts at the experimental endpoint. Data represent mean ± SD.****P* < 0.001; *****P* < 0.0001; statistical significance was determined by two-way ANOVA followed by Šídák’s multiple-comparisons test (B, D) and one-way ANOVA followed by Šídák’s multiple-comparisons test (E).

**
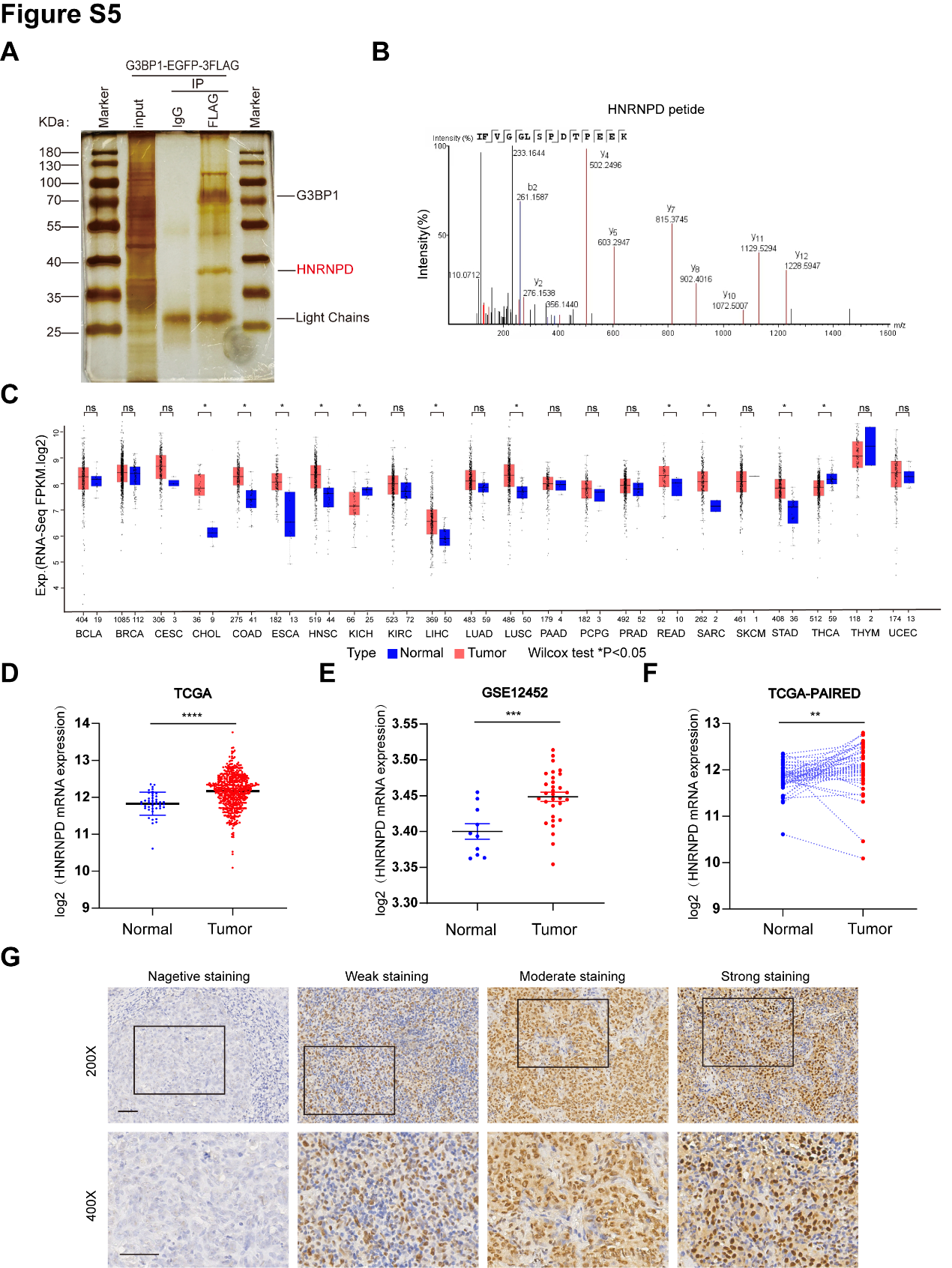
**

**Figure S5. HNRNPD is upregulated in radioresistant nasopharyngeal carcinoma.**(A) Representative silver-stained gel images of proteins immunoprecipitated using anti-IgG or anti-Flag magnetic beads from NPC cell lines overexpressing G3BP1-EGFP-3Flag.(B) Mass spectrometry (MS) identification of HNRNPD.(C) Analysis of HNRNPD expression across multiple cancer types from The Cancer Genome Atlas (TCGA) database.(D) *HNRNPD* mRNA expression levels in normal versus tumor tissues from the TCGA head and neck squamous cell carcinoma (HNSC) dataset.(E) *HNRNPD* mRNA expression levels in normal versus tumor tissues from the GSE12452 dataset.(F) *HNRNPD* mRNA expression in paired normal versus tumor tissues from TCGA HNSC samples.(G) Representative images and scoring of HNRNPD protein expression based on IHC staining intensity in 164 NPC tissue specimens. Scale bar, 50 μm.ns, not significant; **P* < 0.05, ***P* < 0.01, ****P* < 0.001, *****P* < 0.0001; statistical significance was determined by two-sided unpaired Student’s *t*-test (D, E) and two-sided paired Student’s *t*-test (F). TCGA, The Cancer Genome Atlas; HNSC, head and neck squamous cell carcinoma.

**
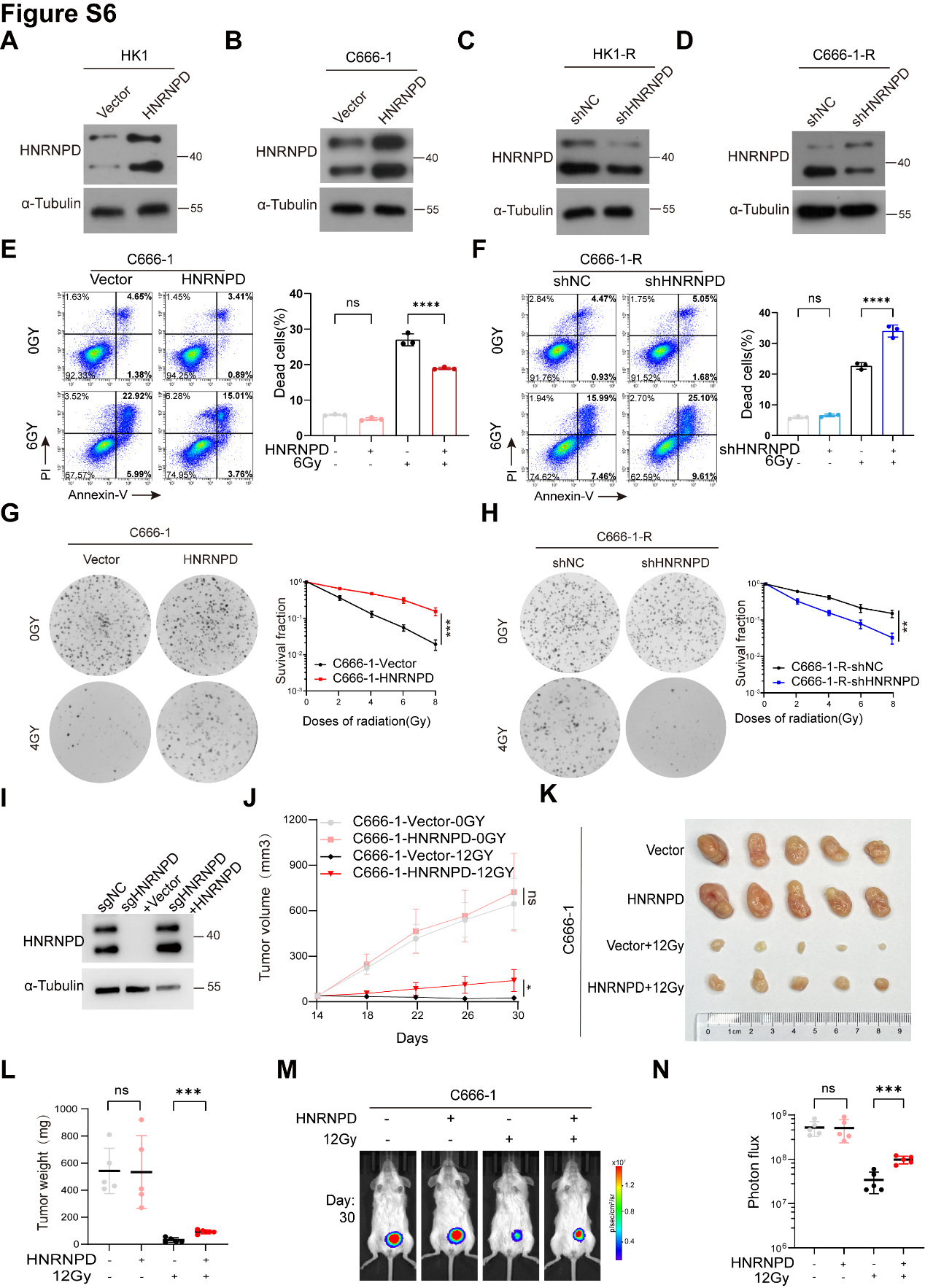
**

**Figure S6. HNRNPD promotes radioresistance in NPC cells *in vitro* and *in vivo*.** (A–D) Western blot analysis of HNRNPD expression in HK1 and C666-1 cells stably overexpressing HNRNPD, and in HK1-R and C666-1-R cells with stable knockdown of HNRNPD.(E, F) Left, representative flow cytometry plots of apoptosis assays. Right, quantified percentages of apoptotic cells in C666-1-Vector vs. C666-1-HNRNPD (E) and C666-1-R-shNC vs. C666-1-R-shHNRNPD (F) cells at 48 h after treatment with or without 6 Gy irradiation. Data represent mean ± SD from three independent experiments.(G, H) Left, representative images of colony formation assays. Right, clonogenic survival curves for C666-1-Vector vs. C666-1-HNRNPD (G) and C666-1-R-shNC vs. C666-1-R-shHNRNPD (H) cells exposed to the indicated radiation doses. Data represent mean ± SD from three independent experiments.(I) Immunoblot analysis confirming HNRNPD knockout and re-expression in HK1-R cells transduced with sgNC, sgHNRNPD+Vector, or sgHNRNPD+HNRNPD. α-Tubulin served as a loading control.(J)Growth curves of C666-1 xenograft tumors in mice receiving the indicated treatments (RT, 2 Gy/fraction, 6 fractions). Data represent mean ± SD (*n* = 5 mice per group).(K) Representative images of excised tumors at the experimental endpoint (day 30).(L) Tumor weights at day 30. Data represent mean ± SD (*n* = 5 tumors per group).(M) Representative *in vivo* bioluminescence images of tumors from the indicated groups on day 30.(N) Quantification of total photon flux from (M). Data represent mean ± SD (*n* = 5).ns, not significant; **P* < 0.05, ***P* < 0.01, ****P* < 0.001, *****P* < 0.0001; statistical significance was determined by one-way ANOVA followed by Šídák’s multiple-comparisons test (E, F, L, N) and two-way ANOVA followed by Šídák’s multiple-comparisons test (G, H, J).

**
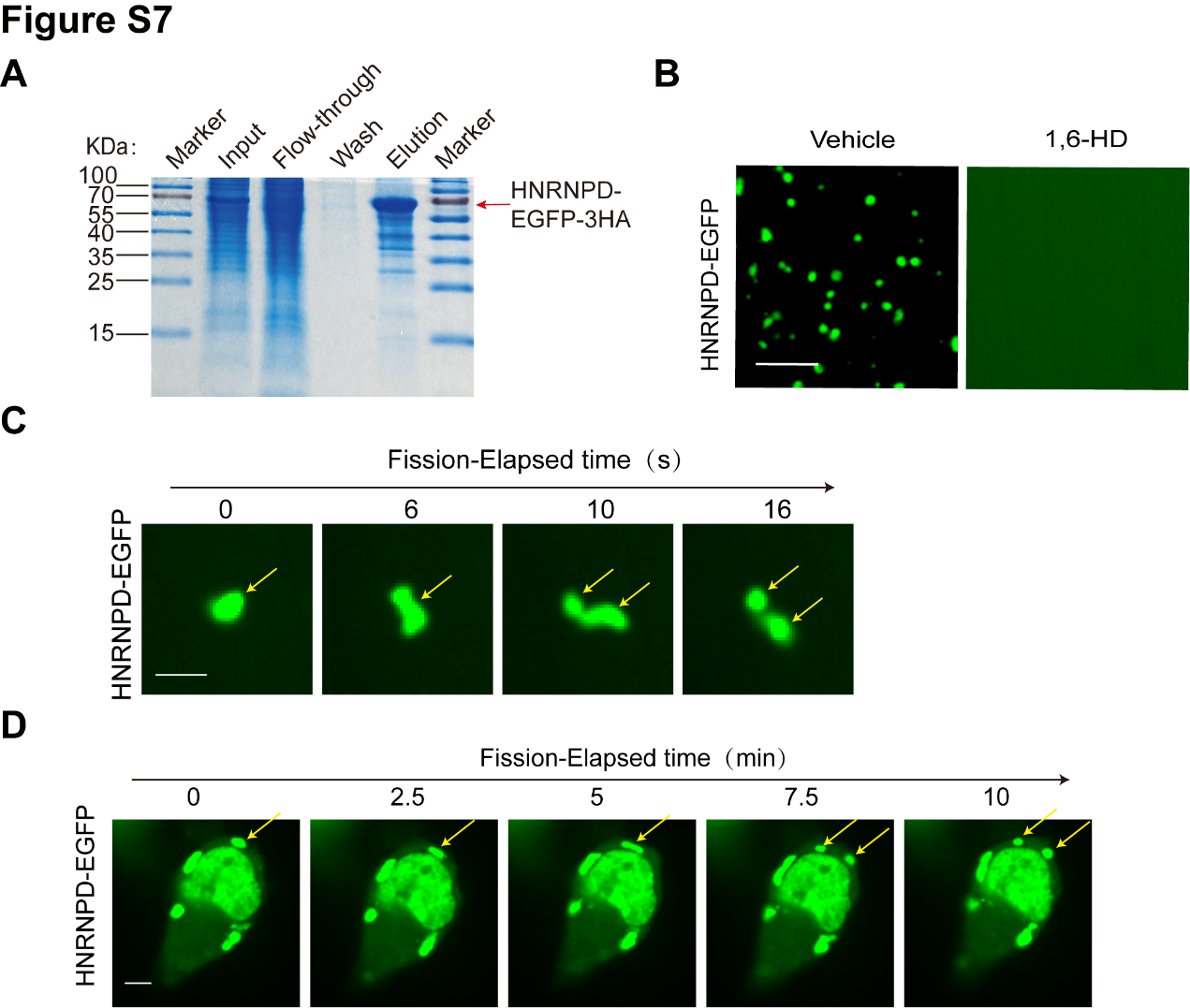
**

**Figure S7. HNRNPD forms biomolecular condensates via its C-terminal IDR.**(A) Coomassie Brilliant Blue staining of intermediate and final elution fractions from the purification of recombinant HNRNPD-EGFP-3HA protein from eukaryotic cells.(B) Effect of 1,6-hexanediol (1,6-HD) treatment on *in vitro* droplet formation of HNRNPD-EGFP-3HA.(C) Time-lapse images showing fission events of HNRNPD-EGFP-3HA droplets *in vitro*. Scale bar, 2 μm.(D) Time-lapse images showing fission behavior of HNRNPD-EGFP condensates in live C666-1-R cells after 6 Gy irradiation. Scale bar, 5 μm.


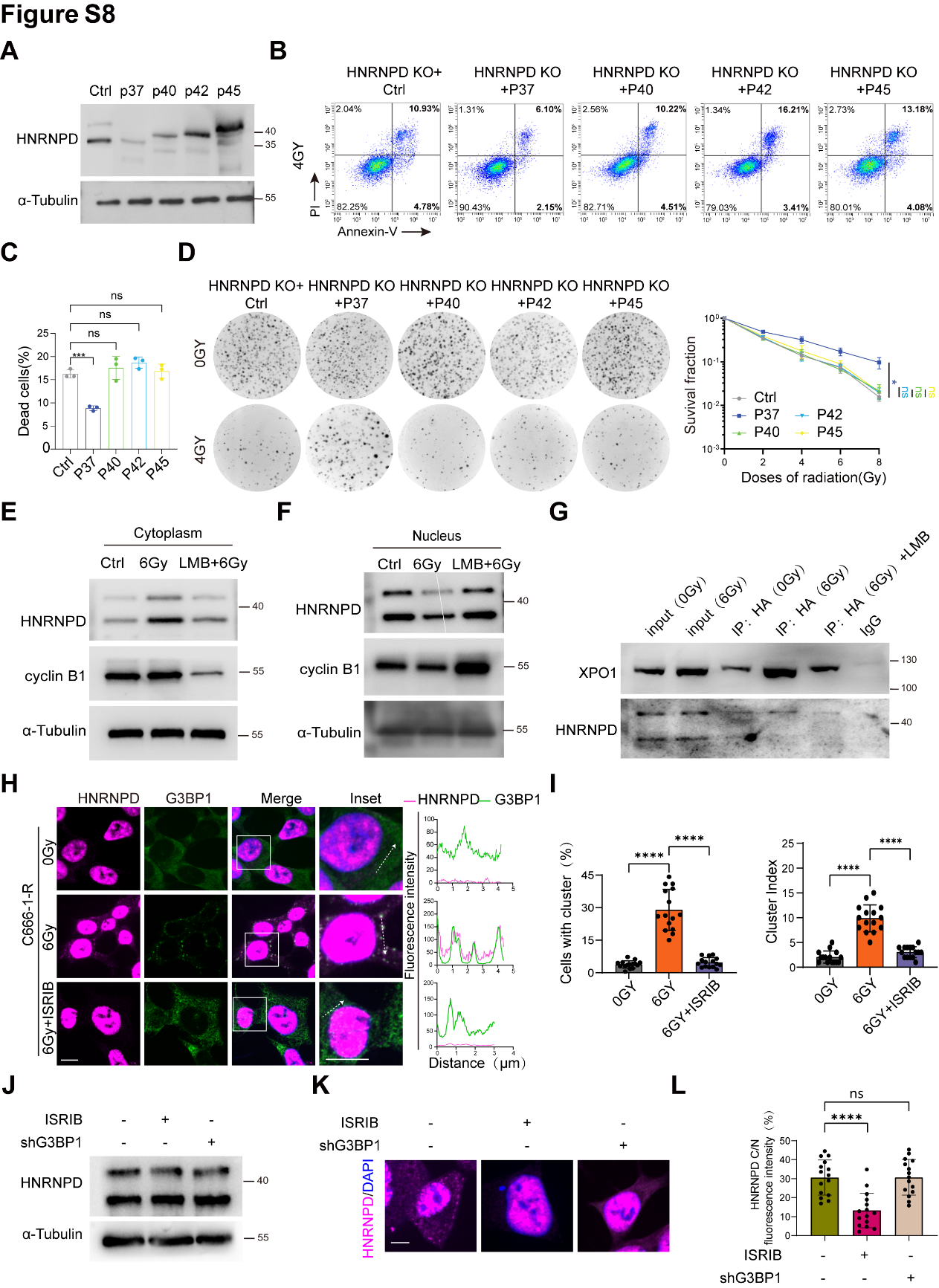


**Figure S8. The p37 isoform is the functionally dominant HNRNPD isoform, and irradiation promotes XPO1-dependent nuclear export of HNRNPD associated with SG formation.**(A) Immunoblot analysis comparing the electrophoretic mobility of endogenous HNRNPD bands in untreated HK1-R cells (Ctrl) with individually re-expressed untagged p37, p40, p42, and p45 isoforms in HNRNPD-knockout HK1-R cells. α-Tubulin served as a loading control.(B, C) Representative flow cytometry plots (B) and quantified percentages of dead cells (C) in HNRNPD-knockout HK1-R cells reconstituted with empty vector or the indicated HNRNPD isoforms at 72 h after 4 Gy irradiation. Data represent mean ± SD from three independent experiments.(D) Left, representative images of colony formation assays in HNRNPD-knockout HK1-R cells reconstituted with empty vector or the indicated HNRNPD isoforms after 0 Gy or 4 Gy irradiation. Right, clonogenic survival curves after exposure to the indicated radiation doses. Data represent mean ± SD from three independent experiments.(E, F) Immunoblot analysis of HNRNPD in the cytoplasmic (E) and nuclear (F) fractions of HK1-R cells under the indicated treatments. Cells were left untreated, exposed to 6 Gy irradiation, or treated with leptomycin B (LMB) before irradiation. Cyclin B1 and α-Tubulin were used as indicated fractionation controls.(G) Co-immunoprecipitation analysis of the interaction between HNRNPD and XPO1 in cells expressing HA-tagged HNRNPD under the indicated conditions. Cell lysates were immunoprecipitated with anti-HA antibody or control IgG and immunoblotted for XPO1 and HNRNPD.(H) Representative confocal images showing colocalization of HNRNPD (magenta) and G3BP1 (green) in C666-1-R cells under the indicated conditions. Right, fluorescence intensity profiles along the indicated line. Scale bar, 5 μm. Insets show magnified views. Inset scale bar, 5 μm.(I) Quantification of the percentage of cells with SG clusters and the SG cluster index from (H). Data are expressed as mean ± SD (*n* = 15 image fields per condition from three independent experiments).(J) Immunoblot analysis of total HNRNPD protein levels in HK1-R cells treated with ISRIB or shG3BP1 under irradiation conditions. α-Tubulin served as a loading control.(K) Representative confocal images showing the subcellular localization of HNRNPD (magenta) in HK1-R cells treated with ISRIB or shG3BP1 under irradiation conditions. Nuclei were stained with DAPI (blue). Scale bar, 5 μm.(L) Quantification of the cytoplasmic-to-nuclear fluorescence intensity ratio of HNRNPD from (K). Data are expressed as mean ± SD.ns, not significant; ****P* < 0.001; *****P* < 0.0001; statistical significance was determined by one-way ANOVA followed by Dunnett’s multiple-comparisons test (C, L), one-way ANOVA followed by Šídák’s multiple-comparisons test (I), and two-way ANOVA followed by Šídák’s multiple-comparisons test (D).


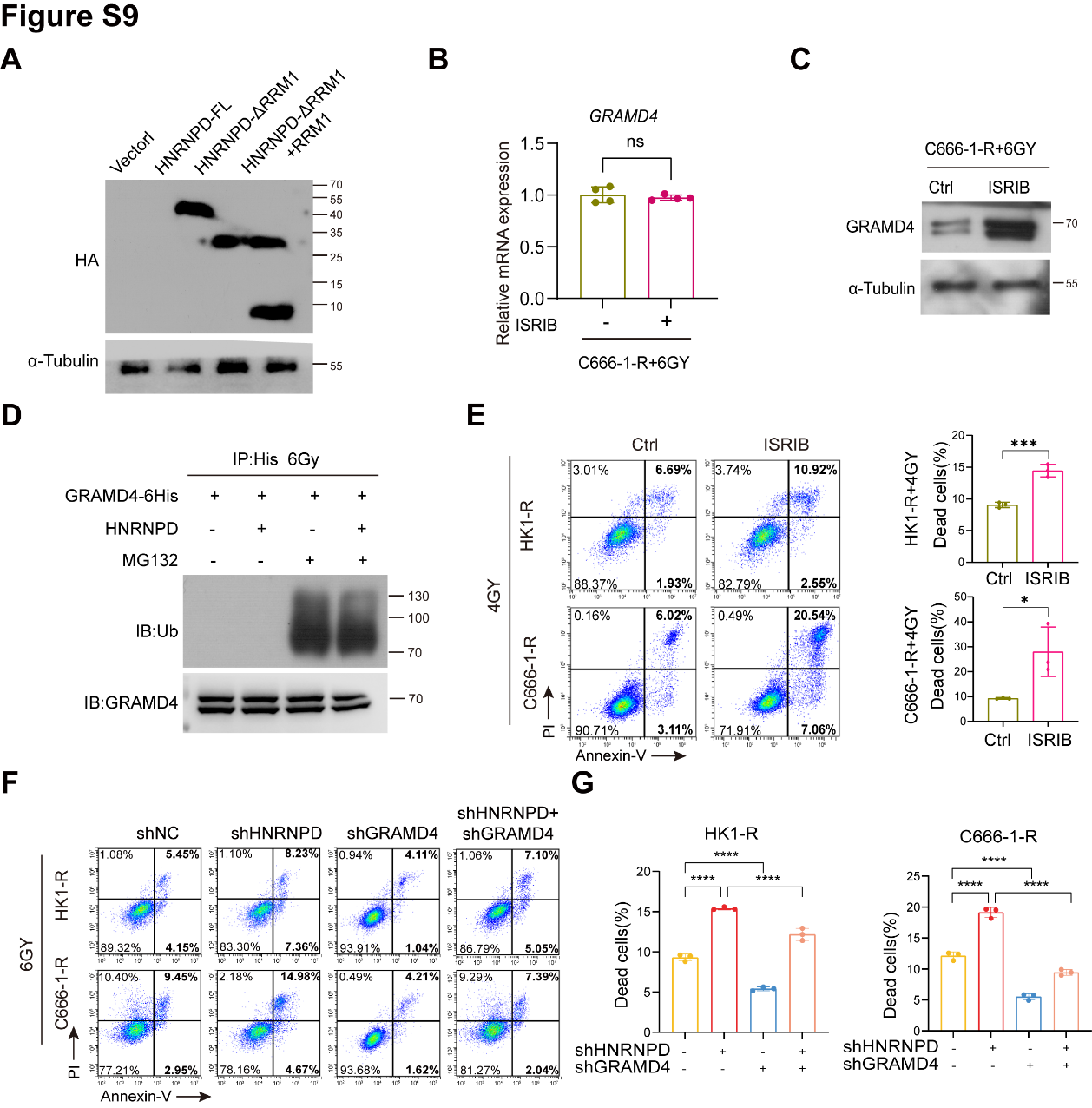


**Figure S9. GRAMD4 contributes to ISRIB-induced and HNRNPD depletion-induced cell death in radioresistant NPC cells.**(A) Immunoblot analysis confirming the expression of HA-tagged full-length HNRNPD (HNRNPD-FL), HNRNPD-ΔRRM1, and the RRM1-complemented HNRNPD-ΔRRM1 construct in HK1-R cells. α-Tubulin served as a loading control.(B) qRT-PCR analysis of GRAMD4 mRNA levels in C666-1-R cells treated with or without ISRIB after 6 Gy irradiation (*n* = 4 biological replicates).(C) Western blot analysis of GRAMD4 protein levels in C666-1-R cells treated with or without ISRIB after 6 Gy irradiation.(D) Ubiquitination assay of GRAMD4 in cells expressing His-tagged GRAMD4 with or without HNRNPD in the presence or absence of MG132 after 6 Gy irradiation. His-tagged GRAMD4 was immunoprecipitated and immunoblotted for ubiquitin and GRAMD4.(E) Representative flow cytometry plots and quantified percentages of dead cells in HK1-R and C666-1-R cells treated with or without ISRIB after 4 Gy irradiation. Data represent mean ± SD from three independent experiments.(F) Representative flow cytometry plots of cell death in HK1-R and C666-1-R cells stably transduced with the indicated shRNAs after 6 Gy irradiation.(G) Quantified percentages of dead cells from (F). Data represent mean ± SD from three independent experiments.ns, not significant; **P* < 0.05; ****P* < 0.001; *****P* < 0.0001; statistical significance was determined by two-sided unpaired Student’s *t*-test (B, E) and one-way ANOVA followed by Šídák’s multiple-comparisons test (G).


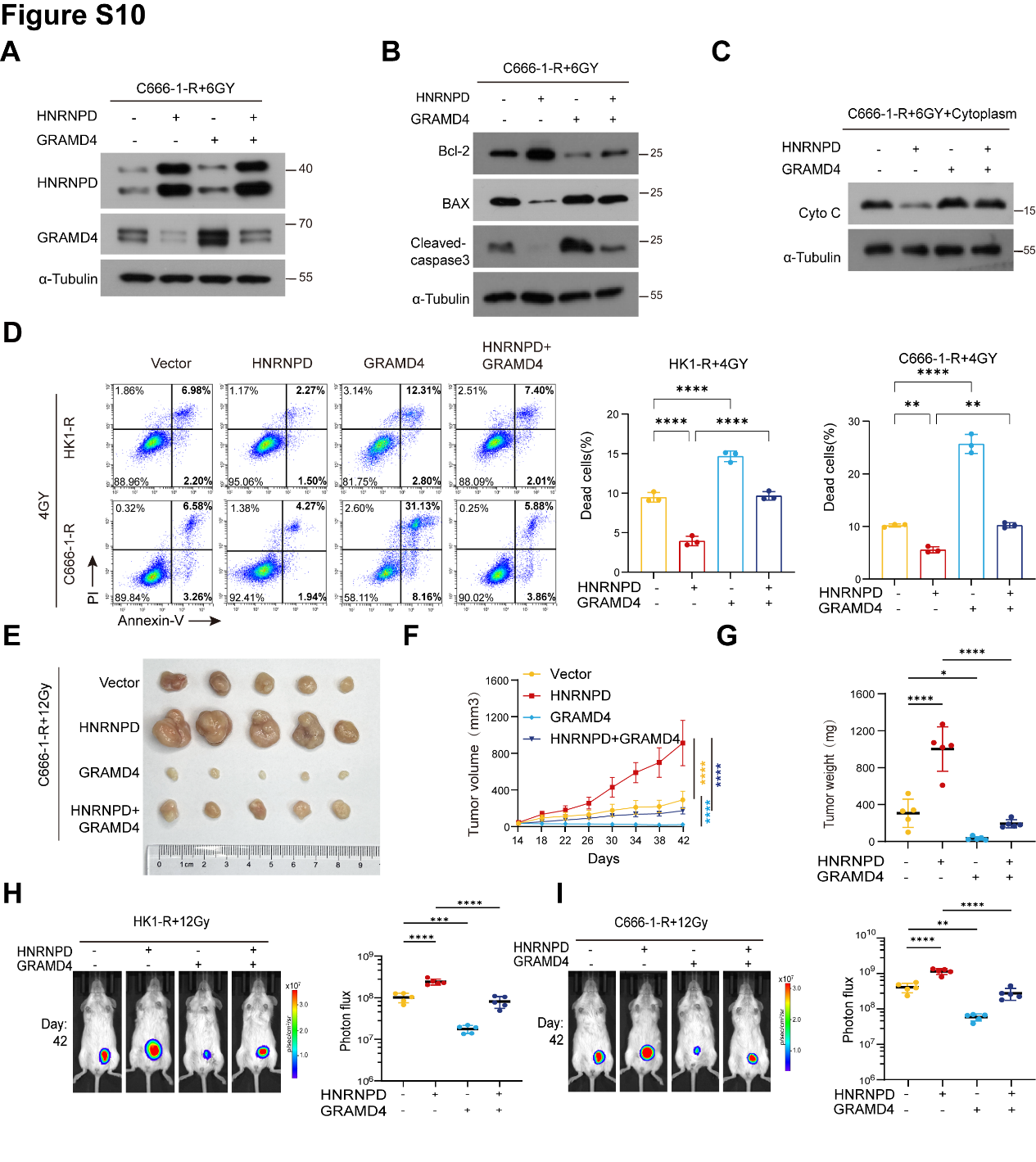


**Figure S10. HNRNPD enhances radioresistance by downregulating GRAMD4 *in vitro* and *in vivo*.**(A) Western blot analysis confirming HNRNPD and GRAMD4 protein expression in C666-1-R cells stably transduced with the indicated constructs after 6 Gy irradiation.(B) Western blot analysis of Bcl-2, BAX, and cleaved caspase-3 levels in C666-1-R cells stably transduced with the indicated constructs after 6 Gy irradiation.(C) Western blot analysis of cytochrome c (Cyto C) release in the cytosolic fraction of C666-1-R cells stably transduced with the indicated constructs after 6 Gy irradiation.(D) Left, representative flow cytometry plots of cell death in HK1-R and C666-1-R cells stably transduced with the indicated constructs after 4 Gy irradiation. Right, quantification of cell death. Data represent mean ± SD from three independent experiments.(E) Representative images of excised tumors from C666-1-R xenografts at the experimental endpoint after radiotherapy (total dose, 12 Gy).(F) Tumor growth curves of C666-1-R xenografts in mice receiving the indicated treatments. Data represent mean ± SD (*n* = 5 mice per group).(G) Tumor weights of C666-1-R xenografts at the experimental endpoint. Data represent mean ± SD (*n* = 5 tumors per group).(H) Left, representative *in vivo* bioluminescence images of HK1-R xenograft tumors at the experimental endpoint after radiotherapy (total dose, 12 Gy). Right, quantification of total photon flux. Data represent mean ± SD (*n* = 5).(I) Left, representative *in vivo* bioluminescence images of C666-1-R xenograft tumors at the experimental endpoint after radiotherapy (total dose, 12 Gy). Right, quantification of total photon flux. Data represent mean ± SD (*n* = 5).**P* < 0.05; ***P* < 0.01; ****P* < 0.001; *****P* < 0.0001; statistical significance was determined by one-way ANOVA followed by Šídák’s multiple-comparisons test (D, G, H, I) and two-way ANOVA followed by Šídák’s multiple-comparisons test (F).

**Supplementary tables**

**Table S1. Clinicopathological characteristics and tumor-specific expression of HNRNPD in NPC.**

| **Parameters** | **N (%)** |
| --- | --- |
| **Sex** |  |
| Male | 124 (75.6) |
| Female | 40 (24.4) |
| **Age, years** |  |
| ≤48 | 86 (52.4) |
| >48 | 78 (47.6) |
| **T classification** |  |
| T1-2 | 43 (26.2) |
| T3-4 | 121 (73.8) |
| **N classification** |  |
| N0-2 | 130 (79.3) |
| N3 | 34 (20.7) |
| **Clinical stage** |  |
| I-II | 89 (54.3) |
| III-IV | 75 (45.7) |
| **Locoregional recurrence** |  |
| No | 141 (86.0) |
| Yes | 23 (14.0) |
| **Distant metastasis** |  |
| No | 124 (75.6) |
| Yes | 40 (24.4) |
| **Vital status** |  |
| Alive | 85 (51.8) |
| Dead | 79 (48.2) |
| **HNRNPD expression** |  |
| Low | 96 (58.5) |
| High | 68 (41.5) |
|  |  |

Abbreviations: T, tumor; N, node; HNRNPD, Heterogeneous Nuclear Ribonucleoprotein D.

**Table S2. Relationship between HNRNPD expression and patient clinicopathological features.**

| Factors | HNRNPD expression | | *P*-value |
| --- | --- | --- | --- |
|  | Low (%) | High (%) |  |
| Sex |  |  |  |
| Male | 71 (43.3) | 53 (32.3) |  |
| Female | 25 (15.2) | 15 (9.1) | 0.558 |
| Age, years |  |  |  |
| ≤48 | 47 (28.7) | 39 (23.8) |  |
| >48 | 49 (29.9) | 29 (17.7) | 0.289 |
| T classification |  |  |  |
| T1-2 | 27 (16.5) | 16 (9.8) |  |
| T3-4 | 69 (42.1) | 52 (31.7) | 0.510 |
| N classification |  |  |  |
| N0-2 | 79 (48.2) | 51 (31.1) |  |
| N3 | 17 (10.4) | 17 (10.4) | 0.256 |
| Clinical stage |  |  |  |
| I-II | 56 (34.1) | 33 (20.1) |  |
| III-IV | 40 (24.4) | 35 (21.3) | 0.214 |
| Locoregional recurrence |  |  |  |
| No | 88 (53.7) | 53 (32.3) |  |
| Yes | 8 (4.9) | 15 (9.1) | 0.065 |
| Distant metastasis |  |  |  |
| No | 75 (45.7) | 49 (29.9) |  |
| Yes | 21 (12.8) | 19 (11.6) | 0.373 |
| Vital status |  |  |  |
| Alive | 55 (33.5) | 30 (18.3) |  |
| Dead | 41 (25.0) | 38 (23.2) | 0.096 |

**Table S3.** **Univariate and multivariate analysis of factors associated with OS and PFS and LRRFS in 164 NPC patients**

| **Characteristics** | **Univariate analysis** | | **Multivariate analysis** | |
| --- | --- | --- | --- | --- |
|  | **HR (95% CI)** | ***P*** | **HR (95% CI)** | ***P*** |
| **OS** |  |  |  |  |
| HNRNPD (High vs. Low) | 1.685(1.082-2.624) | **0.021** | 1.591(1.017-2.490) | **0.042** |
| Gender(Male vs Female) | 1.504(0.857-2.639) | 0.155 |  |  |
| Age (> 48 vs. ≤48) | 1.739(1.112-2.722) | **0.015** | 1.633(1.037-2.569) | **0.034** |
| T category (T3-4 vs. T1-2) | 2.984(1.536-5.796) | **0.001** | 2.232(1.115-4.467) | **0.023** |
| N category (N3 vs. N0-2) | 2.102(1.290-3.423) | **0.003** | 1.204(0.689-2.104) | 0.513 |
| Clinical Stage（III-IV vs. I-II） | 3.040(1.905-4.853) | **<0.001** | 2.151(1.240-3.730) | **0.006** |
| **PFS** |  |  |  |  |
| HNRNPD (High vs. Low) | 1.825(1.182-2.818) | **0.007** | 1.738(1.117-2.704) | **0.014** |
| Gender(Male vs Female) | 1.404(0.813-2.426) | 0.223 |  |  |
| Age (> 48 vs. ≤48) | 1.712(1.102-2.658) | **0.017** | 1.620(1.035-2.537) | **0.035** |
| T category (T3-4 vs. T1-2) | 2.859(1.514-5.400) | **0.001** | 2.141(1.097-4.179) | **0.026** |
| N category (N3 vs. N0-2) | 2.065(1.274-3.349) | **0.003** | 1.198(0.685-2.096) | 0.527 |
| Clinical Stage（III-IV vs. I-II） | 2.859(1.820-4.492) | **<0.001** | 1.997(1.163-3.427) | **0.012** |
| **LRRFS** |  |  |  |  |
| HNRNPD (High vs. Low) | 1.790(1.153-2.779) | **0.010** | 1.759(1.129-2.740) | **0.013** |
| Gender(Male vs Female) | 1.495(0.852-2.621) | 0.161 |  |  |
| Age (> 48 vs. ≤48) | 1.784(1.142-2.785) | **0.011** | 1.686(1.074-2.646) | **0.023** |
| T category (T3-4 vs. T1-2) | 3.084(1.589-5.987) | **0.001** | 2.331(1.168-4.651) | **0.016** |
| N category (N3 vs. N0-2) | 2.025(1.245-3.293) | **0.004** | 1.163(0.667-2.028) | 0.594 |
| Clinical Stage（III-IV vs. I-II） | 2.978(1.877-4.727) | **<0.001** | 2.128(1.234-3.669) | **0.007** |

HR, hazard ratio; CI, confidence interval; OS，overall survival; PFS, progression-free survival; LRRFS, locoregional recurrence-free survival.

**• KEY RESOURCES TABLE**

| REAGENT or RESOURCE | SOURCE | IDENTIFIER |  |  |
| --- | --- | --- | --- | --- |
| **Antibodies** | | |  |  |
| Rabbit polyclonal anti-hnRNP D | Abcam | ab61193, RRID:AB_2117320 |  |  |
| Mouse monoclonal anti-G3BP (for G3BP1 detection) | BD Biosciences | 611126, RRID:AB_398437 |  |  |
| Rabbit monoclonal anti-HA-Tag | Cell Signaling Technology | 3724, RRID:AB_1549585 |  |  |
| DYKDDDDK-Tag (rabbit mAb) | Cell Signaling Technology | 14793T; RRID:AB_2572291 |  |  |
| GRAMD4 (rabbit mAb) | Abcam | ab234649 |  |  |
| Bcl-2 (rabbit mAb) | Abcam | ab182858; RRID:AB_2715467 |  |  |
| BAX (rabbit mAb) | Abcam | ab32503; RRID:AB_725631 |  |  |
| Cleaved caspase-3 (rabbit mAb) | Cell Signaling Technology | 9664T; RRID:AB_2070042 |  |  |
| Cytochrome c (rabbit mAb) | Cell Signaling Technology | 11940S; RRID:AB_2637071 |  |  |
| α-Tubulin (mouse mAb) | Cell Signaling Technology | 3873S; RRID:AB_1904178 |  |  |
| Histone H3 (rabbit mAb) | Cell Signaling Technology | 4499T; RRID:AB_10544537 |  |  |
| HRP-conjugated anti-rabbit IgG | Cell Signaling Technology | 7074S; RRID:AB_2099233 |  |  |
| HRP-conjugated anti-mouse IgG | Cell Signaling Technology | 7076S; RRID:AB_330924 |  |  |
| Alexa Fluor(R) 488 Goat anti-mouse IgG (minimal x-reactivity) | BioLegend | 405319, RRID:AB_2563044 |  |  |
| Goat anti-Rabbit IgG (H+L) Cross-Adsorbed Secondary Antibody, Alexa Fluor™ 647 | Thermo Fisher Scientific | A-21244, RRID:AB_2535812 |  |  |
| Anti-rabbit IgG light chain-specific | Jackson ImmunoResearch | 211-032-171; RRID:AB_2339148 |  |  |
| USP10 (D7A5) Rabbit Monoclonal Antibody | Cell Signaling Technology | 8501; RRID:AB_10949976 |  |  |
| eIF4G Antibody | Cell Signaling Technology | 2498; RRID:AB_2096025 |  |  |
| anti-TIA1 antibody | Abcam | ab140595; RRID:AB_2687963 |  |  |
| anti-EDC4 antibody | Abcam | ab72408; RRID:AB_1268717 |  |  |
| Exportin-1/CRM1 (D6V7N) Rabbit Monoclonal Antibody | Cell Signaling Technology | 46249; RRID:AB_2799298 |  |  |
| Phospho-eIF2 alpha (Ser51) Antibody | Cell Signaling Technology | 9721S; RRID:AB_330951 |  |  |
| eIF2 alpha Antibody | Cell Signaling Technology | 9722S; RRID:AB_2230924 |  |  |
| Cyclin B1 Rabbit mAb | Beyotime | AF1606; RRID:N/A |  |  |
| Ubiquitin (E4I2J) Rabbit mAb | Cell Signaling Technology | 43124S; RRID:AB_2799235 |  |  |
| PE anti-human CD45 Antibody | BioLegend | 304008; RRID:AB_314396 |  |  |
| APC/Cyanine7 anti-human CD8 Antibody | BioLegend | 344714; RRID:AB_2044006 |  |  |
| BD Horizon BV421 Mouse Anti-Human Granzyme B | BD Biosciences | 563389; RRID:AB_2738175 |  |  |
| **Chemicals, peptides, and recombinant proteins** | | |  |  |
| ISRIB | MedChemExpress | HY-12495 |  |  |
| HA Peptide | Beyotime | P9808 |  |  |
| PEG-8000 | HUAYUN | HS1038 |  |  |
| Proteinase K | HUAYUN | HB080 |  |  |
| Blasticidin S | HUAYUN | HY-B9300 |  |  |
| Protease Inhibitor Cocktail | Sigma-Aldrich | P8340 |  |  |
| Phosphatase Inhibitor Cocktail | Sigma-Aldrich | P5726, P0044 |  |  |
| Crystal Violet | Beyotime | C0121 |  |  |
| DAPI | Solarbio | C0065 |  |  |
| Goat Serum Blocking Solution | ZSGB-Bio | ZLI-9056 |  |  |
| EDTA Antigen Retrieval Solution | ZSGB-Bio | ZLI-9069 |  |  |
| SYBR Green qPCR Master Mix | ESscience | QP002 |  |  |
| Anti-Fade Fluorescence Mounting Medium | Abcam | ab104135 |  |  |
| In situ hybridization fixative | Servicebio | G1113 |  |  |
| Prehybridization buffer | Servicebio | G3046 |  |  |
| 2× SSC | Servicebio | G3015 |  |  |
| Collagenase IV | Yeasen | 40510ES60 |  |  |
| DNase I | Roche | 10104159001 |  |  |
| Lipofectamine 3000 | Thermo Fisher Scientific | L3000015 |  |  |
| Leptomycin B | Solarbio | L7940-10ug |  |  |
| Puromycin Dihydrochloride | MP | 0219453925-25mg |  |  |
| MG132 | Sigma-Aldrich | M8699-1MG |  |  |
| IP lysis buffer | Beyotime | P0013 |  |  |
| **Critical commercial assays** | | |  |  |
| RNA Immunoprecipitation Kit | Bersinbio | Bes5101 |  |  |
| Nuclear and Cytoplasmic Protein Extraction Kit | Beyotime | P0027 |  |  |
| Annexin V-AF647/PI Apoptosis Detection Kit | GOONIE | 100-102 |  |  |
| RNA Quick Purification Kit | ESscience | RN001 |  |  |
| Fast Reverse Transcription Kit | ESscience | RT001 |  |  |
| Zombie Aqua Fixable Viability Kit | BioLegend | 423101 |  |  |
| BD Cytofix/Cytoperm solution | BD Biosciences | 554722 |  |  |
| BD Perm/Wash Buffer | BD Biosciences | 554723 |  |  |
| Pierce™ Anti-HA Magnetic Beads | Thermo Scientific | 88836 |  |  |
| Anti-DYKDDDDK Magnetic Agarose | Thermo Scientific | A36797 |  |  |
| Pierce Ni-NTA Magnetic Agarose Beads | Thermo Scientific | 78605 |  |  |
| Anti-IgG magnetic beads | Elabscience | EA-IP-100M |  |  |
| **Experimental models: Cell lines** |  |  |  |  |
| HK1 (NPC cell line) | Tong Xiang, Sun Yat-sen University Cancer Center | RRID:CVCL_7084 |  |  |
| C666-1 (NPC cell line) | Tong Xiang, Sun Yat-sen University Cancer Center | RRID:CVCL_7949 |  |  |
| HEK293T | Tong Xiang, Sun Yat-sen University Cancer Center | RRID:CVCL_0063 |  |  |
| **Experimental models** |  |  |  |  |
| NCG mice, female, 4-week-old | Gempharmatech | N/A |  |  |
| Human peripheral blood mononuclear cells (PBMCs) | Healthy donor, this study | N/A |  |  |
| **Oligonucleotides** | | |  |  |
| GRAMD4 FISH probe (488-labeled, sequence: 5'-GCTCGTAGAACCACTTCTGCAGGCCAAAGTTAGTGACCGGCTTGGCACGGCACGATGCTCCACTGTATCCGCGGCTGGACCCACATGAACAAGTTCCCTCCAGATGATATAGGGCGTGTC-3’) | Servicebio | N/A |  |  |
| shG3BP1 (5′-CCCGTAAGAAGGAATGTTA-3′) | Umine Biotechnology | N/A |  |  |
| shHNRNPD (5′-AAUGUUGGUCUUAGUAAAUGT-3′) | Umine Biotechnology | N/A |  |  |
| shGRAMD4 (5′-GCAATTTCCACGAGATCTTCACTCGAGTGAAGATCTCGTGGAAATTGC-3′) | Umine Biotechnology | N/A |  |  |
| sgHNRNPD (5′-CCCTTCGGTGCCTCCAGACG-3′) | Umine Biotechnology | N/A |  |  |
| sgG3BP1 (5′-GCTCATGCCACGCTAAATGA-3′) | Umine Biotechnology | N/A |  |  |
| qRT-PCR primers *HNRNPD* Fwd: GGCTCCTGCCACCTGCTAAT; Rev: CAGAGGGACCCAACGTCATAC | RiboBio | N/A |  |  |
| qRT-PCR primers *G3BP1* Fwd: GTGCTGCGGGTTAGGTGAT; Rev: GTGCTCCTTGCTACATGGATG | RiboBio | N/A |  |  |
| qRT-PCR primers *GRAMD4* Fwd: GTGGGACTCTATGCTGGTATCAA; Rev: TATAGGGCGTGTCGTACTTGG | RiboBio | N/A |  |  |
| qRT-PCR primers *GAPDH* Fwd: GAACGGGAAGCTCACTGG; Rev: GCCTGCTTCACCACCTTCT | RiboBio | N/A |  |  |
| **Recombinant DNA** |  |  |  |  |
| pcDNA3.1(+)-3HA-EGFP-hHNRNPD | Umine Biotechnology | N/A |  |  |
| 3HA-tagged HNRNPD isoforms (p37,p40,p42,p45) | Umine Biotechnology | N/A |  |  |
| untagged HNRNPD isoforms (p37,p40,p42,p45) | Umine Biotechnology | N/A |  |  |
| HNRNPD truncation mutants (2–97, 1–180, 98–257, 181–355, 258–355) | Umine Biotechnology | N/A |  |  |
| HNRNPD ΔIDR mutants (ΔIDR-C, ΔIDR-N) | Umine Biotechnology | N/A |  |  |
| HNRNPD ΔRRM mutants (ΔRRM, ΔRRM1, RRM1, ΔRRM2) | Umine Biotechnology | N/A |  |  |
| 3Flag-tagged G3BP1 | Umine Biotechnology | N/A |  |  |
| 6His-tagged GRAMD4 | Umine Biotechnology | N/A |  |  |
| pLV-CMV-hHNRNPD-3HA-IRES-Puro | Umine Biotechnology | N/A |  |  |
| pLV-CMV-6His-GRAMD4-PGK-Bla | Umine Biotechnology | N/A |  |  |
| pLKD-U6-shHNRNPD-CMV-EGFP-2A-Puro | Umine Biotechnology | N/A |  |  |
| pLKD-U6-shG3BP1-CMV-EGFP-2A-Puro | Umine Biotechnology | N/A |  |  |
| pLKD-U6-shGRAMD4-CMV-EGFP-2A-Blasticidin | Umine Biotechnology | N/A |  |  |
| pLenti-U6-sgHNRNPD-EFS-NS-SpCas9-2A-Puro | Umine Biotechnology | N/A |  |  |
| pLenti-U6-sgG3BP1-EFS-NS-SpCas9-2A-Puro | Umine Biotechnology | N/A |  |  |
| sgRNA-resistant rescue constructs for HNRNPD and G3BP1 | Umine Biotechnology | N/A |  |  |
| **Software and algorithms** |  |  |  |  |
| ImageJ | NIH | RRID:SCR_003070 |  |  |
| NIS-Elements Viewer | Nikon | RRID:SCR_014329 |  |  |
| GraphPad Prism 10 | GraphPad Software | RRID:SCR_002798 |  |  |
| SPSS Statistics 26.0 | IBM | RRID:SCR_016479 |  |  |
| GSEA software | Broad Institute | RRID:SCR_003199 |  |  |
| DESeq2 | Bioconductor | RRID:SCR_015687 |  |  |
| MSigDB database | Broad Institute | RRID:SCR_016863 |  |  |
| Living Image® software | Caliper | RRID:SCR_014247 |  |  |

**Supplementary Methods**

**Co-immunoprecipitation (Co-IP) and Protein Purification**

HK1, C666-1, or HEK293T cells were transfected with tagged plasmids using Lipofectamine 3000 (Thermo Scientific, L3000015) and irradiated as indicated. Whole-cell lysates were prepared with IP lysis buffer (Beyotime, P0013) supplemented with protease inhibitors (Sigma-Aldrich, P8340) and phosphatase inhibitors (Sigma, P5726, P0044). Lysates were incubated overnight at 4 °C with Pierce™ Anti-HA Magnetic Beads (Thermo Scientific, 88836), Anti-DYKDDDDK Magnetic Agarose (Thermo Scientific, A36797), Pierce™ Ni-NTA Magnetic Agarose Beads (Thermo Scientific, 78605),or Anti-IgG magnetic beads (Elabscience, EA-IP-100M). After washing, immunoprecipitated complexes were eluted and subjected to Western blotting, mass spectrometry, or *in vitro* droplet formation assays.

**Plasmids and transient transfection**

Plasmids used for transient expression assays were purchased from Umine Biotechnology. These included 3HA-tagged HNRNPD expression constructs encoding wild-type HNRNPD, truncation mutants (2–97, 1–180, 98–257, 181–355, and 258–355), HNRNPD isoforms (p37, p40, p42, and p45), a 3HA-EGFP-HNRNPD construct, HNRNPD-ΔIDR mutants (ΔIDR-C and ΔIDR-N), and HNRNPD-ΔRRM mutants (ΔRRM, ΔRRM1, RRM1, and ΔRRM2). A 3Flag-tagged G3BP1 construct and a 6His-tagged GRAMD4 construct were also used for transient assays. All constructs were sequence-verified before use. Transient transfection was performed using Lipofectamine 3000 (Thermo Fisher Scientific, L3000015) according to the manufacturer’s instructions. These transiently transfected constructs were used primarily for co-immunoprecipitation, protein interaction, and domain-mapping experiments.

**Lentiviral transduction and generation of stable overexpression/knockdown cell lines**

Lentiviral particles were purchased directly from Umine Biotechnology. Stable overexpression and knockdown cell lines were generated by transducing target cells with lentiviral particles carrying pLV-CMV-hHNRNPD-3HA-IRES-Puro, pLV-CMV-6His-GRAMD4-PGK-Bla, pLKD-U6-shHNRNPD-CMV-EGFP-2A-Puro, pLKD-U6-shG3BP1-CMV-EGFP-2A-Puro, pLKD-U6-shGRAMD4-CMV-EGFP-2A-Blasticidin, or the corresponding control vectors. In addition, untagged HNRNPD isoform constructs (p37, p40, p42, and p45) were used for stable rescue experiments in HNRNPD-deficient cells. The shRNA targeting sequences were as follows: shHNRNPD, 5′-AATGTTGGTCTTAGTAAATGT-3′; shG3BP1, 5′-CCCGTAAGAAGGAATGTTA-3′; and shGRAMD4, 5′-GCAATTTCCACGAGATCTTCACTCGAGTGAAGATCTCGTGGAAATTGC-3′. After transduction, stable cell populations were selected with 2 μg/mL puromycin dihydrochloride (MP, 0219453925) or 5 μg/mL blasticidin S (HUAYUN, HY-B9300), depending on the resistance cassette carried by the vector. Stable overexpression and knockdown efficiency was confirmed by qRT-PCR and Western blotting before subsequent experiments.

**CRISPR/Cas9-mediated knockout and rescue**

HNRNPD- and G3BP1-knockout cells were generated using the CRISPR/Cas9 vector pLenti-U6-sgRNA-EFS-NS-SpCas9-2A-Puro. The sgRNA sequences were as follows: HNRNPD sgRNA, 5′-CCCTTCGGTGCCTCCAGACG-3′; and G3BP1 sgRNA, 5′-GCTCATGCCACGCTAAATGA-3′. The CRISPR/Cas9 plasmids were delivered into the indicated cells, and knockout efficiency was confirmed by Western blotting before subsequent analyses. For rescue experiments, sgRNA-resistant HNRNPD or G3BP1 constructs were purchased from Umine Biotechnology and introduced into the corresponding knockout cells for reconstitution assays.

**Establishment of Radiation-Resistant Cell Lines**

HK1 and C666-1 cells in the exponential growth phase were seeded into T25 flasks. When cells reached 60–70% confluence, they were irradiated with 2 Gy X-rays using X-ray irradiator. Fresh medium was replaced every 2 days. When cell density reached 90%, cells were passaged and exposed to an additional 2 Gy irradiation 24 hours later. Irradiation was repeated until a cumulative dose of 40 Gy (20 sessions) was achieved, and cells were subsequently passaged for 2–3 additional generations to establish radiation-tolerant NPC cell lines HK1-R and C666-1-R. Parental cells were cultured in parallel without irradiation.

**Live-cell imaging**
For *in vitro* droplet imaging, protein solutions containing EGFP-HNRNPD-3HA were added dropwise to confocal petri dishes. For live-cell imaging in cells, EGFP-labeled HNRNPD-transfected HK1 cells or G3BP1-EGFP-transfected HK1-R cells were exposed to irradiation and then seeded into glass-bottomed dishes for time-lapse observation. Live-cell imaging was performed using a rotating disk confocal live cell imager (CSU-W1; Nikon) equipped with an environmental chamber maintained at 37 °C with 5% CO2. Images were acquired at regular intervals using a 100× objective lens for the indicated time periods to monitor the dynamic behavior of HNRNPD condensates or G3BP1-positive stress granules, including their formation, fusion, fission, and dissolution. NIS-Elements Viewer software (Nikon) was used for image acquisition and subsequent processing.

**Western Blotting**

Western blotting was performed as previously described^[1]^. Nuclear and cytoplasmic proteins were extracted using nuclear and cytoplasmic protein extraction kits (Beyotime, P0027), according to the manufacturer’s instructions. α-Tubulin (1:1000, Cell Signaling Technology, 3873S) and Histone H3 (1:1000, Cell Signaling Technology, 4499T) served as cytoplasmic and nuclear loading controls, respectively. In some fractionation experiments, Cyclin B1 (1:1000, Beyotime, AF1606) was used as an additional nuclear marker, as indicated. The following primary antibodies were used: HNRNPD (1:1000, Abcam, ab61193), G3BP1 (1:1000, BD Biosciences, 611126), HA-Tag (1:1000, Cell Signaling Technology, 3724T), DYKDDDDK-Tag (1:1000, Cell Signaling Technology, 14793T), GRAMD4 (1:1000, Abcam, ab234649), Bcl-2 (1:1000, Abcam, ab182858), BAX (1:1000, Abcam, ab32503), Cleaved caspase-3 (1:1000, Cell Signaling Technology, 9664T), cytochrome c (1:1000, Cell Signaling Technology, 11940S), Exportin-1/CRM1 (D6V7N) Rabbit Monoclonal Antibody (1:1000, Cell Signaling Technology, 46249), Phospho-eIF2 alpha (Ser51) Antibody (1:1000, Cell Signaling Technology, 9721S), eIF2 alpha Antibody (1:1000, Cell Signaling Technology, 9722S), Cyclin B1 Rabbit mAb (1:1000, Beyotime, AF1606), and Ubiquitin (E4I2J) Rabbit mAb (1:1000, Cell Signaling Technology, 43124S). The secondary antibodies were HRP-conjugated anti-mouse or anti-rabbit IgG (Cell Signaling Technology, 7076S, 7074S). For immunoprecipitation assays, an anti-rabbit IgG light chain-specific antibody (Jackson ImmunoResearch, 211-032-171) was used to minimize heavy chain interference.

**Immunohistochemistry (IHC) Staining and Evaluation**

IHC was performed as previously described^[2]^, using antibodies against HNRNPD (1:1000, Abcam, ab61193), GRAMD4 (1:200, Abcam, ab234649), Bcl-2 (1:500, Abcam, ab182858), and Cleaved caspase-3 (1:1000, Cell Signaling Technology, 9664T). Two independent pathologists, blinded to clinical data, evaluated staining based on the percentage of positive tumor cells (0–4) and staining intensity (0–3). A staining index (SI) was calculated by multiplying intensity and extent scores; samples with SI ≥ 6 were defined as high expression, and those with SI < 6 as low expression.

**Hematoxylin and eosin (H&E) staining**
Paraffin-embedded sections of the heart, liver, spleen, lung, and kidney were deparaffinized, rehydrated, and stained with hematoxylin and eosin according to standard procedures. The stained sections were then examined histologically to assess potential systemic toxicity of drug treatment.

**Radiation Treatment**

For *in vitro* irradiation, cells were exposed to X-rays using an irradiator (RS2000PRO,225 kV; Rad Source Technologies, USA). For *in vivo* irradiation, anesthetized mice were immobilized in a customized lead-shielded device, with only the subcutaneous tumor region exposed to the radiation field.

**Colony Formation Assay**

Cells were seeded in 6-well plates and irradiated with 0, 2, 4, 6, or 8 Gy X-rays after 24 hours of incubation at 37 °C. After 10–14 days, colonies were washed with PBS, fixed with 4% formaldehyde, and stained with 0.05% crystal violet. Colonies containing at least 50 cells were counted manually. The surviving fraction (SF) was calculated as: SF = (number of colonies) / (number of cells seeded × plating efficiency), where plating efficiency (PE) was defined as the number of colonies in the control group divided by the number of cells seeded. Dose-response curves were fitted using the linear-quadratic model in GraphPad Prism 10.0 (La Jolla, USA).

**RNA sequencing**

Total RNA was extracted using TRIzol for RNA-seq analysis by Gene Denovo Technology Co., Ltd (Guangzhou, China). Raw RNA-seq data were mapped to the human reference genome (Ensembl release 111). Differential gene expression between groups was analyzed using DESeq2 software, followed by Gene Ontology pathway enrichment analysis^[3]^. Gene set enrichment analysis (GSEA) was performed using GSEA software and the MSigDB database.

**Extraction of RNA, Reverse Transcription, and Quantitative Real-Time PCR**

Total RNA was extracted using an RNA Quick Purification Kit (ESscience, RN001) and reverse transcribed into cDNA with a Fast Reverse Transcription Kit (ESscience, RT001). qPCR was performed with SYBR Green qPCR Master Mix (ESscience, QP002) on a ROCHE LightCycler 480 II system (Basel, Switzerland), following the manufacturer’s instructions. The primers used were:

- ***HNRNPD*** forward: 5′-GGCTCCTGCCACCTGCTAAT-3′; reverse: 5′-CAGAGGGACCCAACGTCATAC-3′
- ***G3BP1*** forward: 5′-GTGCTGCGGGTTAGGTGAT-3′; reverse: 5′-GTGCTCCTTGCTACATGGATG-3′
- ***GRAMD4*** forward: 5′-GTGGGACTCTATGCTGGTATCAA-3′; reverse: 5′-TATAGGGCGTGTCGTACTTGG-3′
- ***GAPDH*** forward: 5′-GAACGGGAAGCTCACTGG-3′; reverse: 5′-GCCTGCTTCACCACCTTCT-3′

Gene expression was normalized to *GAPDH* and analyzed using the 2^–ΔCt or 2^–ΔΔCt method. All experiments were performed in quadruplicate.

**Flow Cytometry *in vitro***

Following the indicated treatments, cells were harvested and stained with an Annexin V-AF647/PI Apoptosis Detection Kit (GOONIE, 100-102) according to the manufacturer’s instructions.

***In vivo* flow cytometry analysis of human immune cells in huPBMC-NCG mice:** Peripheral blood samples were collected from huPBMC-NCG mice at the indicated time points to monitor human immune-cell engraftment. The proportion of human CD45+ cells among live cells was determined by flow cytometry. For analysis of tumor-infiltrating immune cells, tumor tissues collected at the experimental endpoint were minced and digested with Collagenase IV (Yeasen, 40510ES60, 2 mg/ml) and DNase I (Roche, 10104159001, 40 U/ml), and then passed through 100 μm cell strainers to obtain single-cell suspensions. Red blood cells were removed using red blood cell lysis buffer. Cells were first stained with Zombie Aqua Fixable Viability Kit (BioLegend, 423101, 1:500) for live/dead discrimination, and simultaneously stained with PE anti-human CD45 antibody (BioLegend, 304008, 1:500) and APC/Cyanine7 anti-human CD8 antibody (BioLegend, 344714, 1:500) for surface markers. Cells were then fixed and permeabilized with BD Cytofix/Cytoperm solution (BD Biosciences, 554722), washed with 1× BD Perm/Wash Buffer (BD Biosciences, 554723), and stained intracellularly with BD Horizon BV421 Mouse Anti-Human Granzyme B antibody (BD Biosciences, 563389, 1:500). Flow cytometry was then performed to analyze immune-cell populations. Gating was sequentially performed as cells, single cells, live cells, human CD45+ cells, and CD8+ cells, followed by analysis of Granzyme B+ cells within the CD8+ population. The percentages of human CD45+ cells among live cells and Granzyme B+ cells among CD8+ cells were calculated.

**References**

[1] SONG M, HE J, PAN Q Z, et al. Cancer-Associated Fibroblast-Mediated Cellular Crosstalk Supports Hepatocellular Carcinoma Progression [J]. Hepatology, 2021, 73(5): 1717-35.

[2] CHEN C L, WANG Y, HUANG C Y, et al. IL-17 induces antitumor immunity by promoting beneficial neutrophil recruitment and activation in esophageal squamous cell carcinoma [J]. Oncoimmunology, 2017, 7(1): e1373234.

[3] CHEN Y, OUYANG D, WANG Y, et al. EBV promotes TCR-T-cell therapy resistance by inducing CD163+M2 macrophage polarization and MMP9 secretion [J]. J Immunother Cancer, 2024, 12(6).
